# Supplementary material for: Copper integrative catalytic pairs with mixed-valence Cu2+-Cu3+ Species for selective alkyne conversion
Source: Nat Commun. 2025 Oct 30;16:9600. doi: 10.1038/s41467-025-64639-w (PMC12575668; doi:10.1038/s41467-025-64639-w)
Supplement: Supplementary file 1 — Supplementary Information [file 41467_2025_64639_MOESM1_ESM.pdf]

Supporting Information

Copper Integrative Catalytic Pairs with Mixed-Valence Cu^{2+}-Cu^{3+} Species for Selective Alkyne Conversion

Yuxue Yue[1], Mingde Yu[1], Zhangyi Yao[2], Guangzong Fang[3], Bolin Wang[4], Saisai Wang[1], Chunxiao Jin[1], Renqin Chang[5], Tulai Sun[1], Zhiyan Pan[6], Yihan Zhu[1,5], Feng Ryan Wang[*2], Xiaonian Li[*1], Jia Zhao[*1]

[1] Institute of Industrial Catalysis of Zhejiang University of Technology, Zhejiang Key Laboratory of Surface and Interface Science and Engineering for Catalysts, Hangzhou, 310014 (China)

[2] Department of Chemical Engineering, University College London, London WC1E 7JE (UK)

[3] State Key Laboratory of Catalysis, Dalian Institute of Chemical Physics Chinese Academy of Science, Zhongshan Road 457, Dalian, 116023 (China)

[4] School of Chemical Engineering, Northeast Electric Power University, Jilin, 132012 (China)

[5] Research Center of Analysis Measurement, Zhejiang University of Technology Hangzhou, 310014 (China)

[6] College of Environment, Zhejiang University of Technology, Hangzhou, 310014 (China)

[*]Corresponding Author. Email: ryan.wang@ucl.ac.uk; xnli@zjut.edu.cn; jiazhao@zjut.edu.cn.

Table of Contents

- Supplemental Notes 3
- Supplementary Figures 5
- Supplementary Tables 40
- Supplementary References 50

Supplemental Notes

Internal and external mass transfer limitations

Internal Diffusion: Weisz-Prater Criterion

The absence of internal mass transfer limitations was evaluated using the Weisz-Prater criterion, where if C_{WP} is lower than 1, the internal mass transfer effects can be neglected:

$$C_{WP} = \frac{-r'_{A(obs)}\rho_c R^2}{D_e C_{AS}} < 1$$

$-r'_{A(obs)} =$ Observed reaction rate: $2.34 \cdot 10^{-4}$ $kmol \cdot s^{-1} kg_{Cat}^{-1}$

ρ_c = Solid catalyst density: 800 $kg^{-3} m^{-3}$

R = Particle radius: $6.25 \cdot 10^{-5}$ m

C_{AS} = Concentration of reaction gas at the surface of the catalyst. C_{AS} = $1.4 \cdot 10^{-2}$ $kmol \cdot m^{-3}$

De = Effective gas-phase diffusity:

$$D_e = \frac{D_{AB}\varepsilon_p \sigma_c}{\tau}$$

D_{AB} = Gas-phase diffusivity. D_{AB} for a mixture was calculated according to Perry's Chemical Engineer's Handbook to be $4.13 \cdot 10^{-5}$ m^2/s

ε_p = Pellet porosity = 0.5, σ_c = Constriction factor = 0.5, τ = Tortuosity = 3, D_e = $0.34 \cdot 10^{-5}$ $m^2 \cdot s^{-1}$

Solving equation (S. 1)

$$C_{WP} = \frac{2.34 \cdot 10^{-4}\ kmol\ s^{-1} \cdot kg_{Cat}^{-1} \cdot 800\ kg_{Cat} m^{-3} \cdot (6.25 \cdot 10^{-5}\ m)^2}{0.34 \cdot 10^{-5}\ m^2 s^{-1} \cdot 4.09 \cdot 10^{-2}\ kmol\ m^{-3}} = 0.014 \ll 1$$

Therefore, this system does not suffer from internal mass transfer limitations.

External Diffusion: Mears Criterion

The absence of external mass transfer limitations can be evaluated using the Mears criterion:

$$\frac{-r'_A \rho_b R n}{k_c C_{Ab}} < 0.15$$

$-r'_{A(obs)} =$ Observed reaction rate: $2.34 \cdot 10^{-4}$ kmol·s^{-1}kg$^{-1}_{Cat}$

ρ_b= bulk density of the catalyst bed: 396 kg·m^{-3}

R = Particle radius: $6.25 \cdot 10^{-5}$ m

n = reaction order If n = 2

C_{Ab} = Concentration of reaction gas at the surface of the catalyst. C_{AS} = $1.4 \cdot 10^{-2}$ kmol·m^{-3}

k_c = mass transfer coefficient (m s^{-1}) = 0.14 m s^{-1}

k_c can be calculated from the Sherwood number

$$Sh = \frac{k_c d_p}{D_A} = 0.91 \cdot 0.91 \cdot Re^{0.49} \cdot Sc^{1/3}$$

Solving equation (S.3):

$$\frac{2.34 \cdot 10^{-4}\ kmol \cdot s^{-1} kg^{-1}_{Cat} \cdot 396 kg_{Cat} m^{-3} \cdot 3 \cdot 10^{-5} m \cdot 2}{0.14 m \cdot s^{-1} \cdot 1.4 \cdot 10^{-2} kmol\ m^{-3}} = 0.0028 \ll 0.15$$

Therefore, this system does not suffer from external mass transfer limitations.

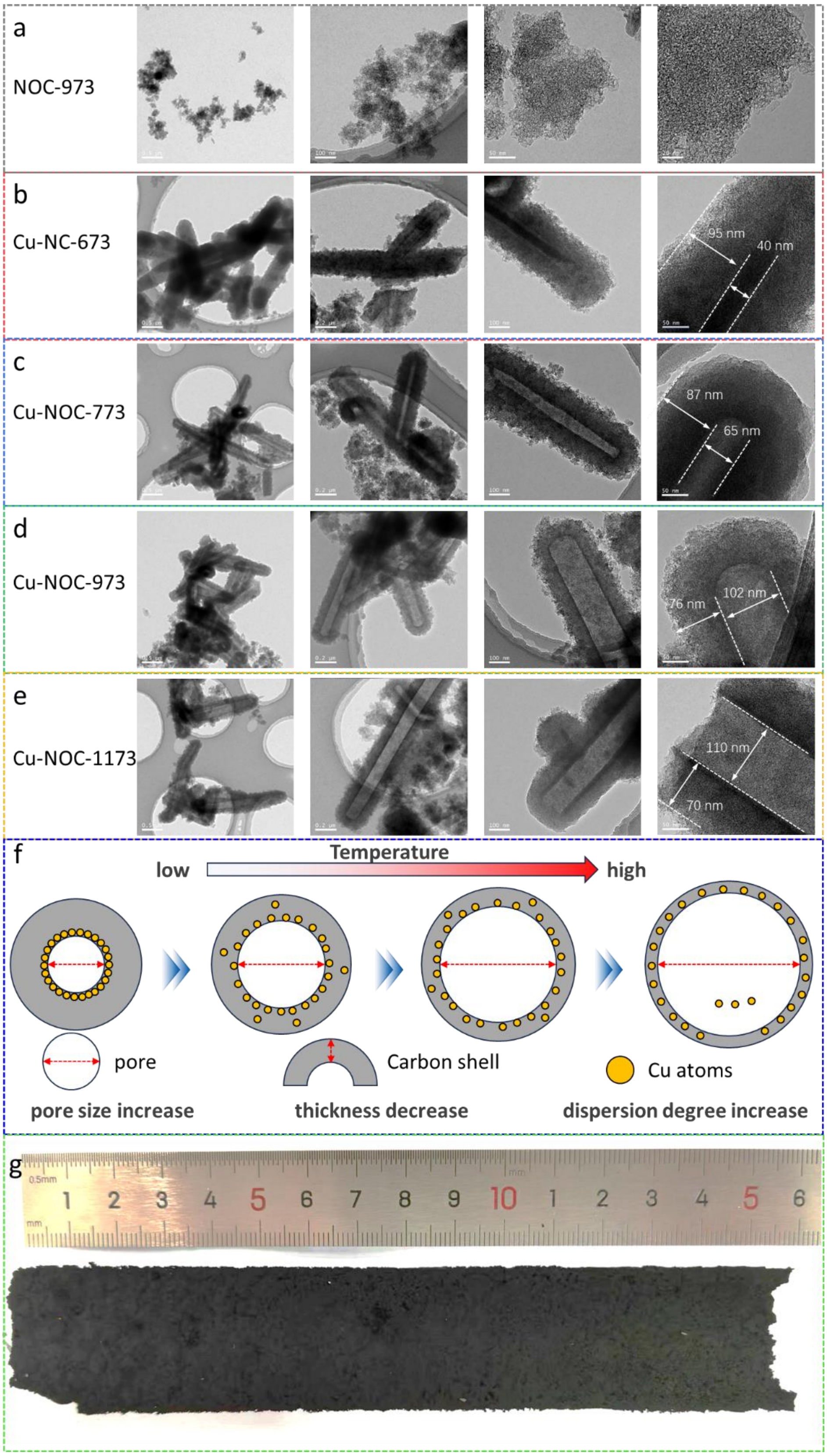

Supplementary Figure 1. TEM images of (a) NOC-973; (b) Cu-NOC-673; (c) Cu-NOC-773; (d) Cu-NOC-973 and (e) Cu-NOC-1173; (f) Schematic diagram of Cu dispersion and pore structure evolution; (g) Photograph of the Cu-NOC-973 sample.

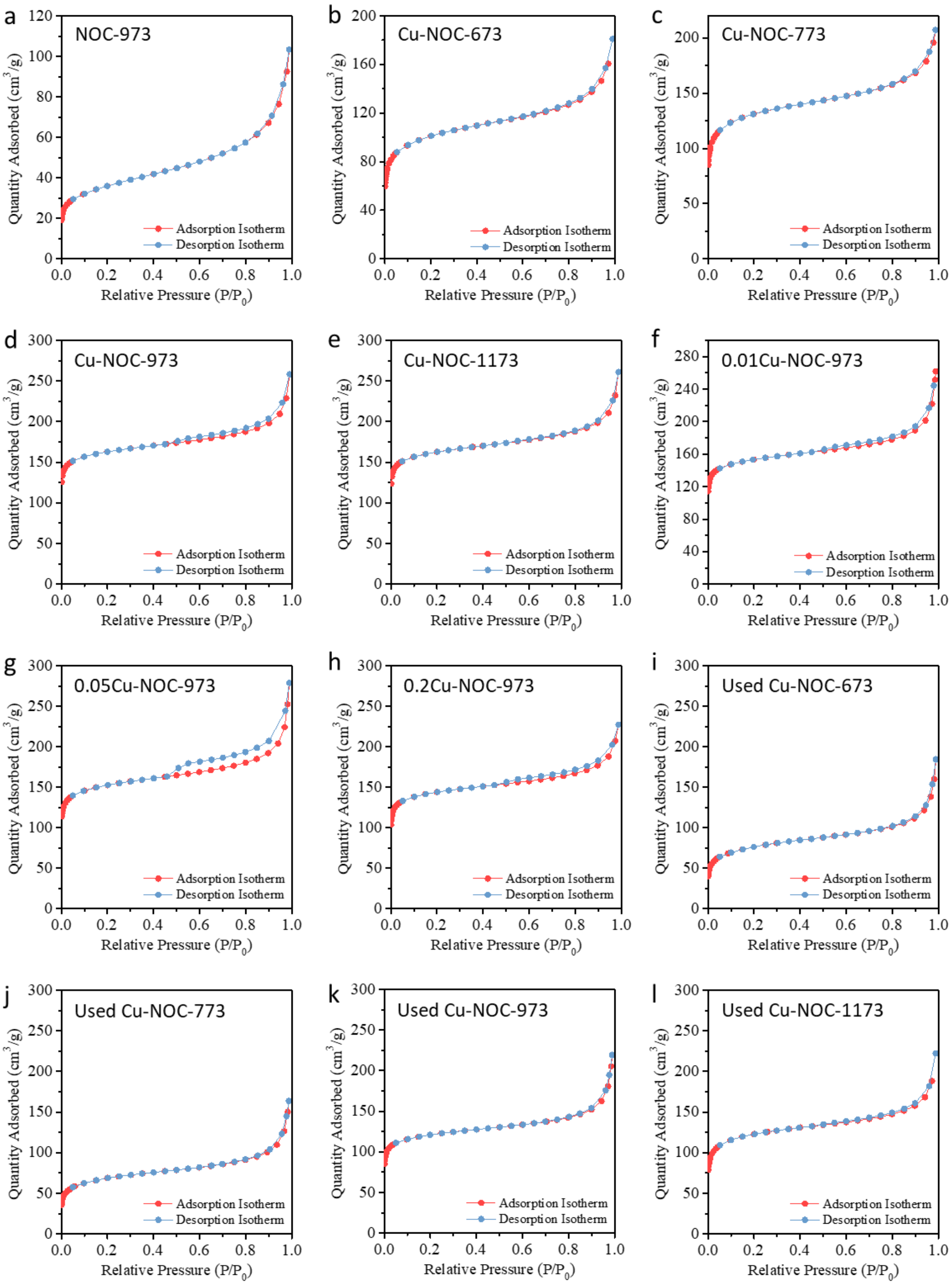

Supplementary Figure 2. N_2 adsorption-desorption isotherm of (a) NOC-973, (b) Cu-NOC-673, (c) Cu-NOC-773, (d) Cu-NOC-973, (e) Cu-NOC-1173, (f) 0.01Cu-NOC-973, (g) 0.05Cu-NOC-973, (h) 0.2Cu-NOC-973, (i) used Cu-NOC-673, (j) used Cu-NOC-773, (k) used Cu-NOC-973, (l) used Cu-NOC-1173 catalysts.

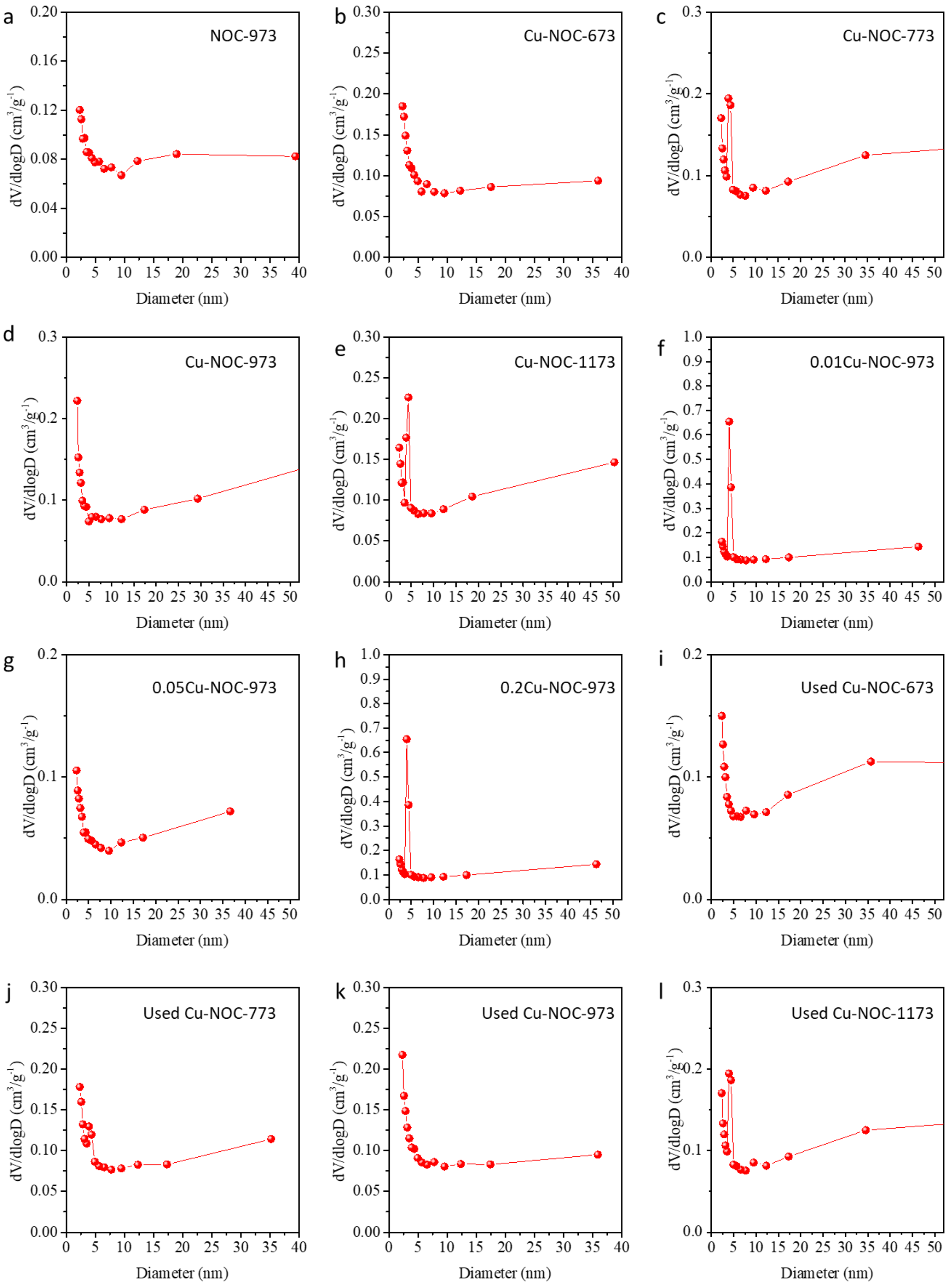

Supplementary Figure 3. Pore distribution of (a) NOC-973, (b) Cu-NOC-673, (c) Cu-NOC-773, (d) Cu-NOC-973, (e) Cu-NOC-1173, (f) 0.01Cu-NOC-973, (g) 0.05Cu-NOC-973, (h) 0.2Cu-NOC-973, (i) used Cu-NOC-673, (j) used Cu-NOC-773, (k) used Cu-NOC-973, (l) used Cu-NOC-1173 catalysts.

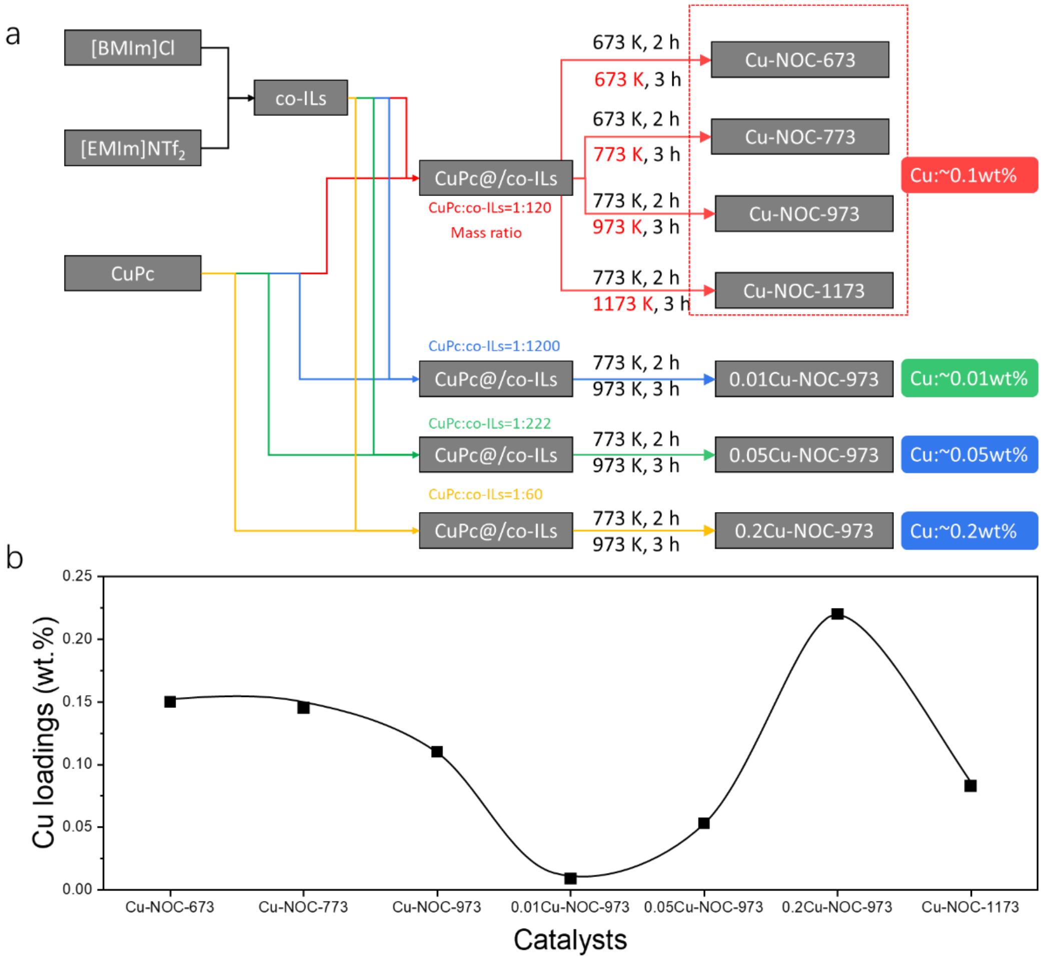

Supplementary Figure 4. (a) List of synthesized Cu-NOC catalysts; (b) the content of Cu in obtained Cu-NOC catalysts (determined by ICP-MS).

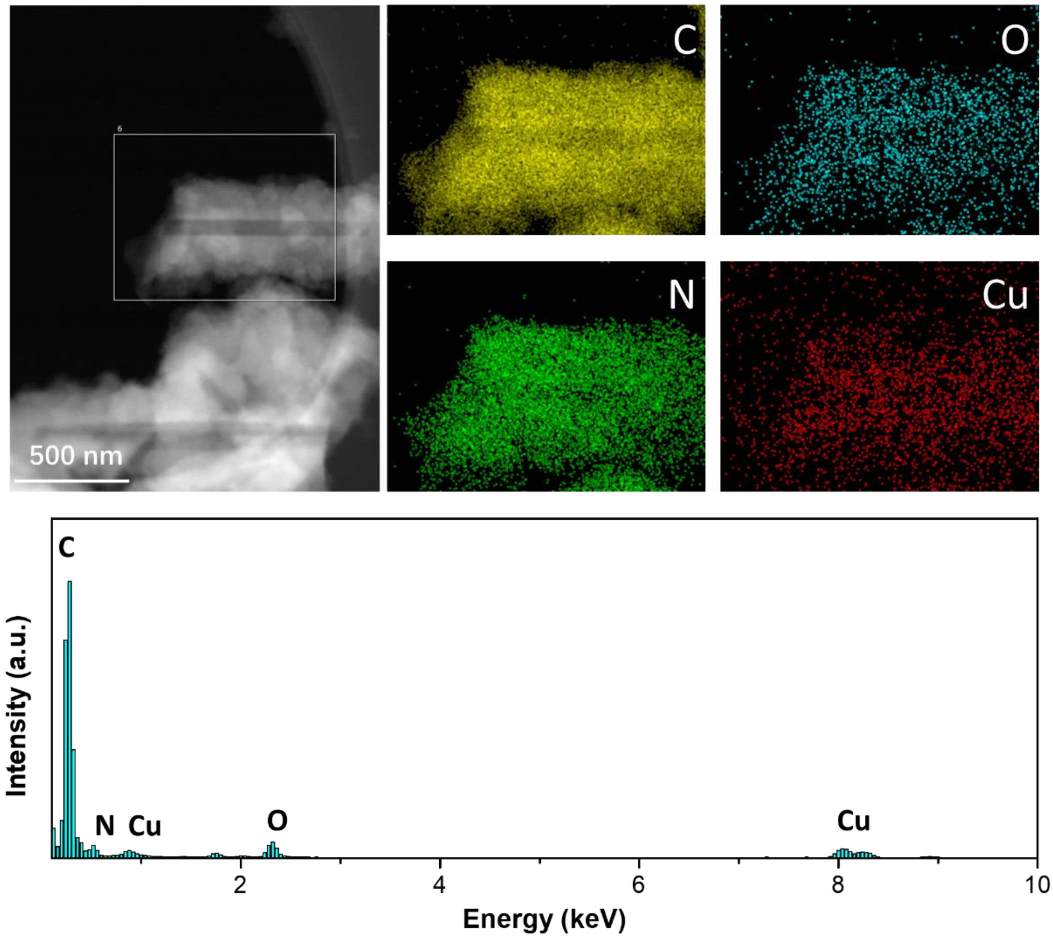

Supplementary Figure 5. Element mapping of Cu-NOC-673.

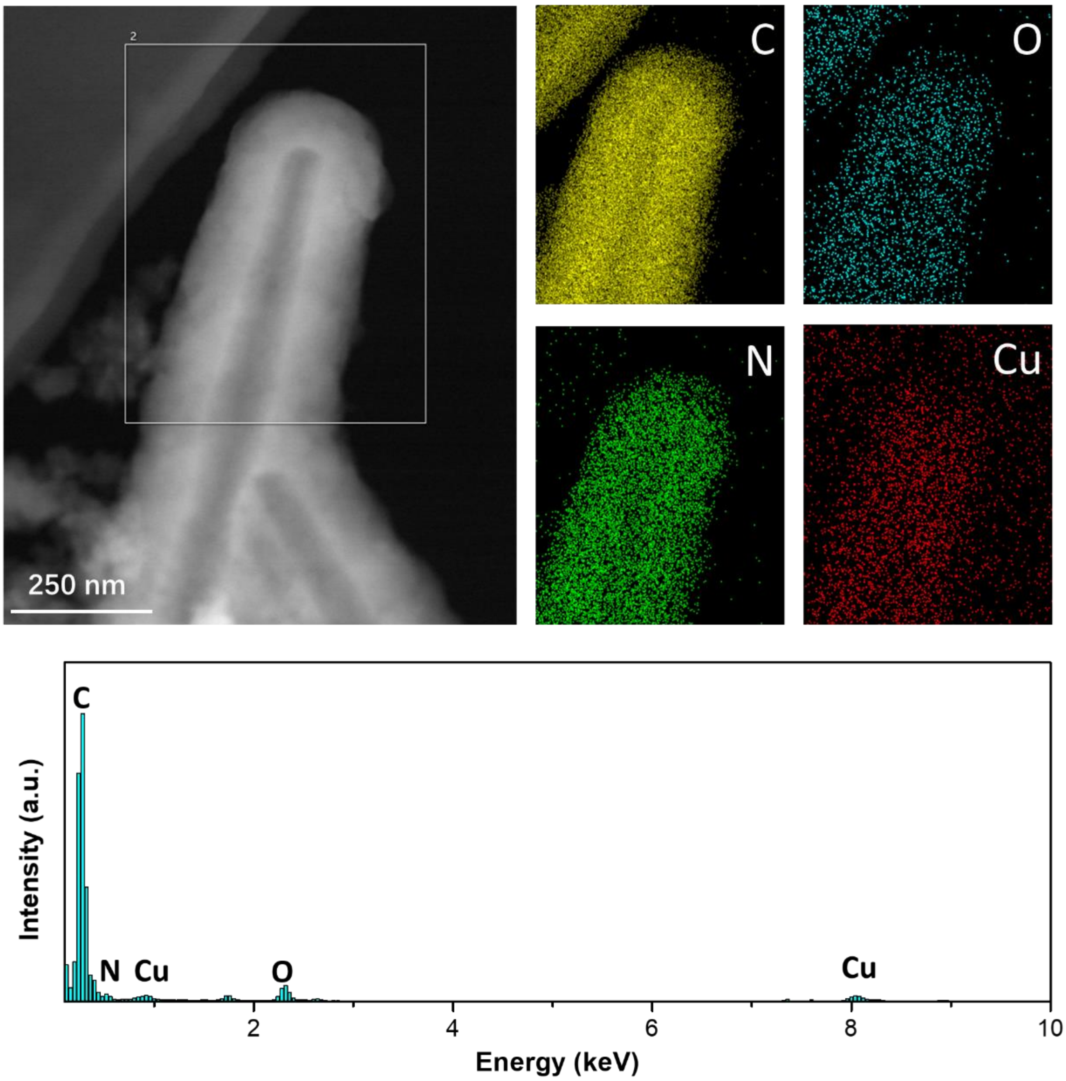

Supplementary Figure 6. Element mapping of Cu-NOC-773.

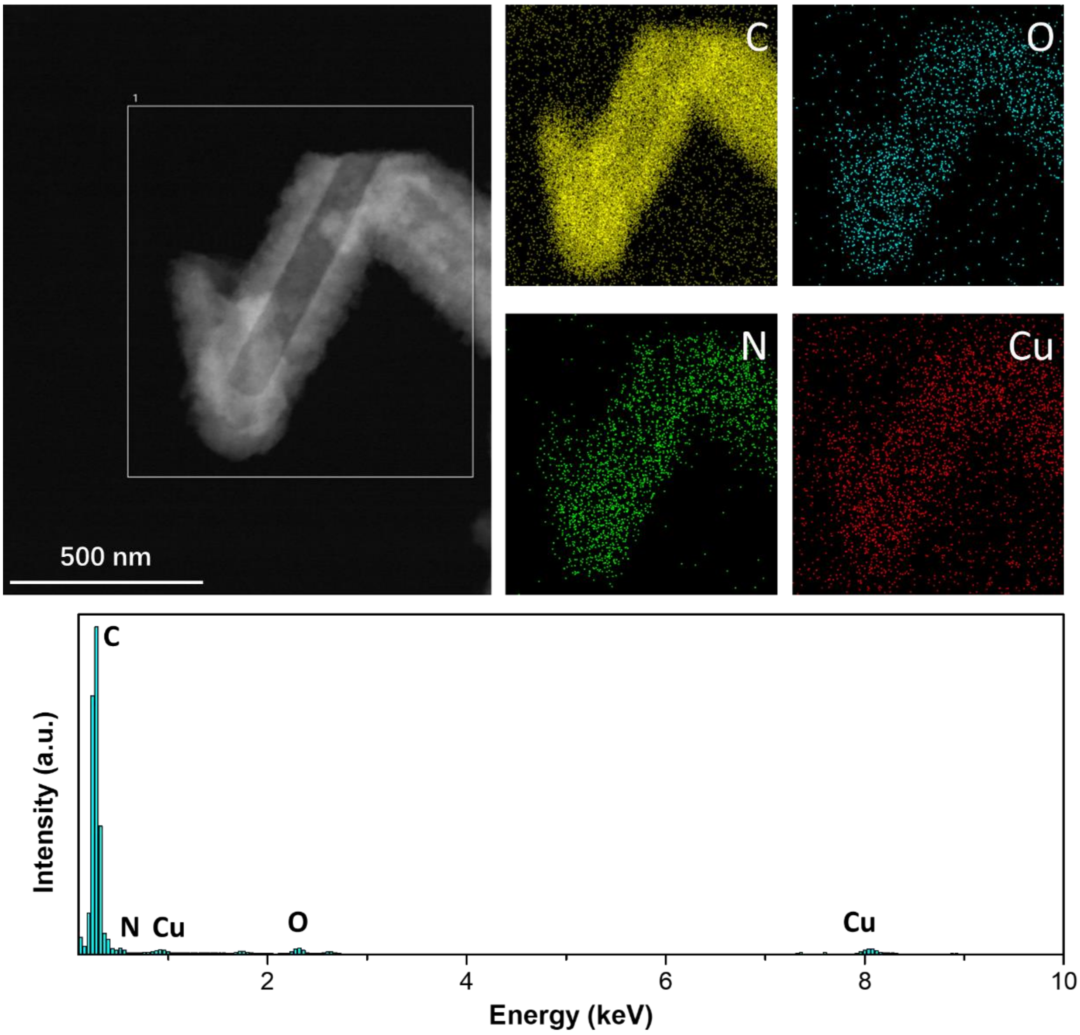

Supplementary Figure 7. Element mapping of Cu-NOC-1173.

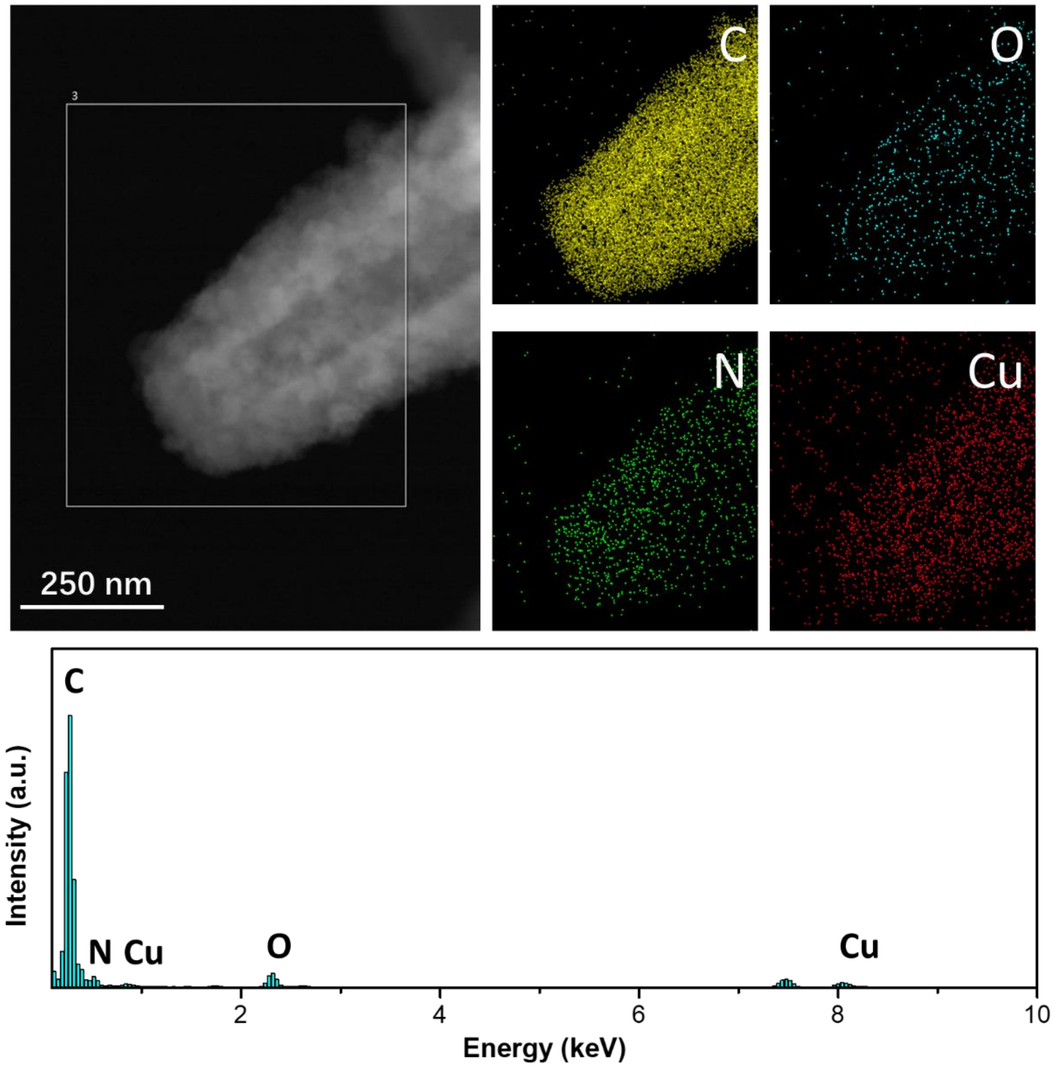

Supplementary Figure 8. Element mapping of 0.01Cu-NOC-973.

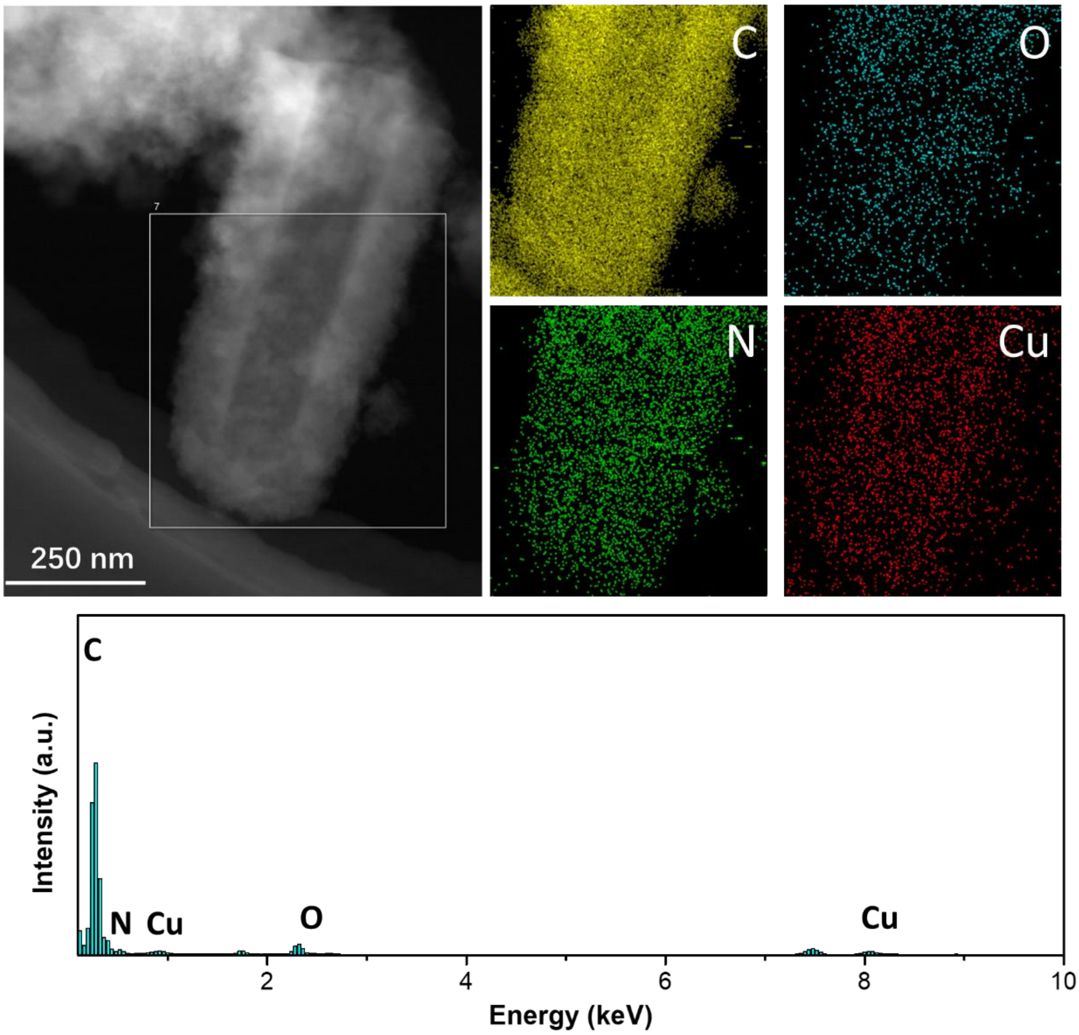

Supplementary Figure 9. Element mapping of 0.05Cu-NOC-973.

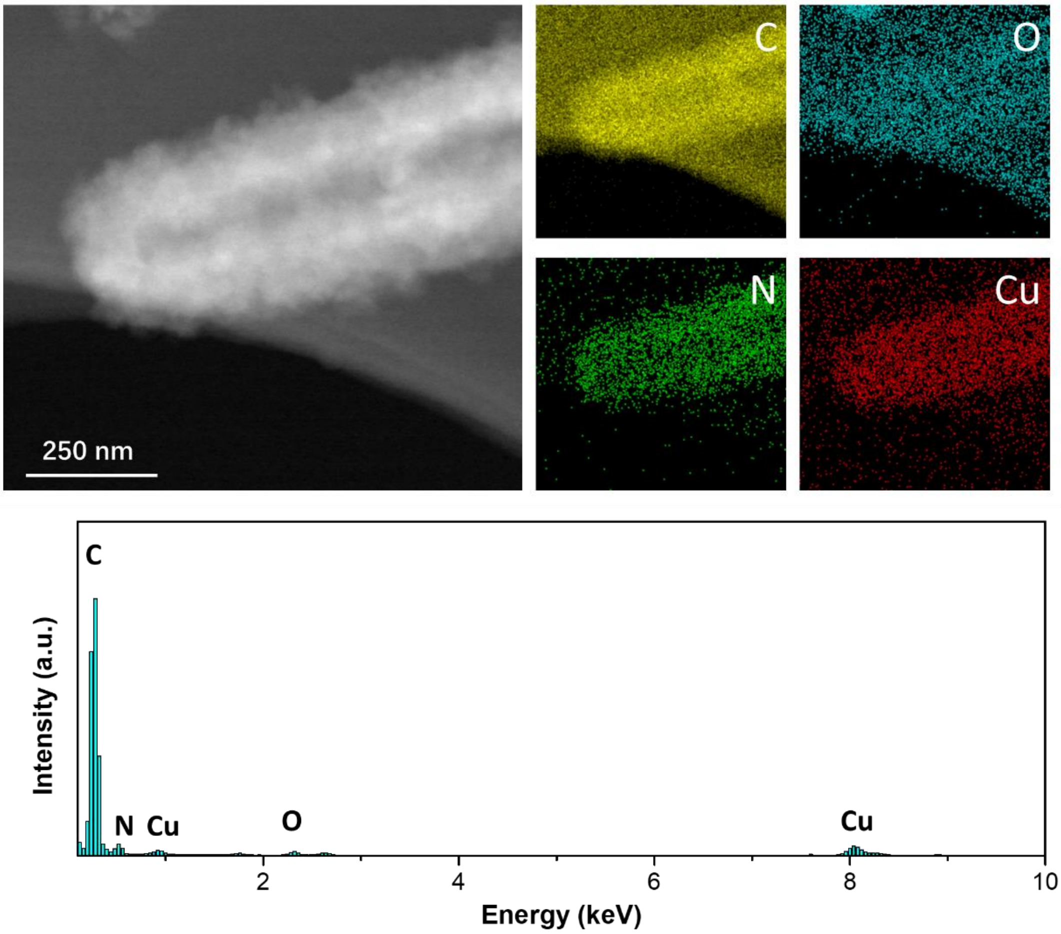

Supplementary Figure 10. Element mapping of 0.2Cu-NOC-973.

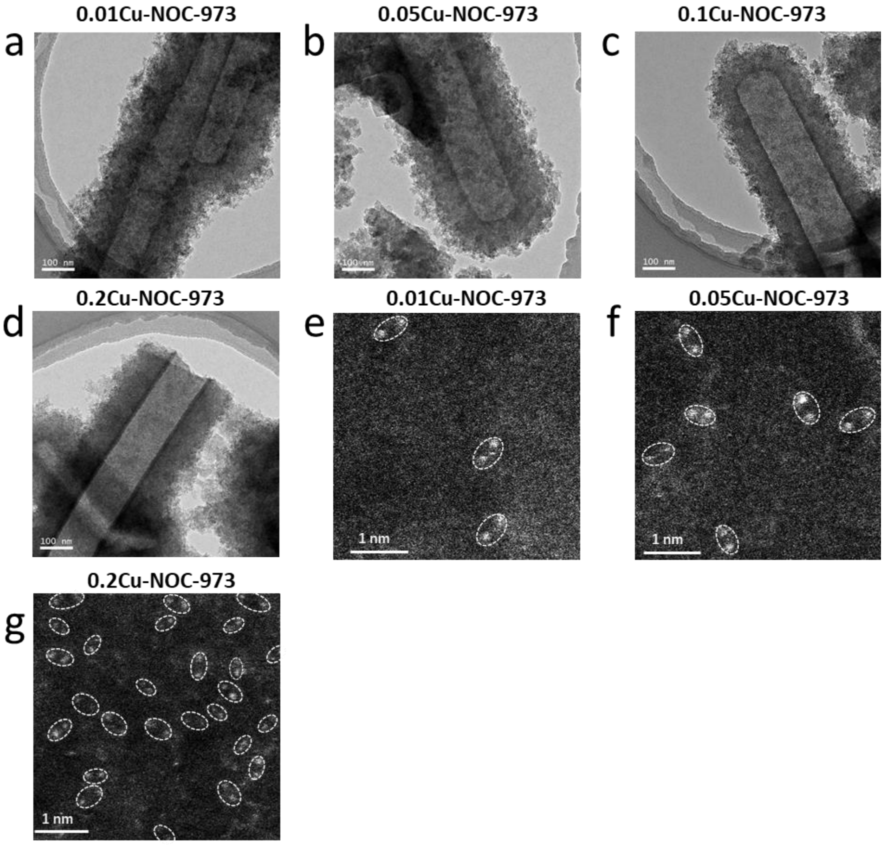

Supplementary Figure 11. TEM images of (a) 0.01Cu-NOC-973, (b) 0.05 Cu-NOC-973, (c) 0.1Cu-NOC-973, (d) 0.2Cu-NOC-973; and HAADF-STEM of (e) 0.01Cu-NOC-973, (f) 0.05 Cu-NOC-973, (g) 0.2Cu-NOC-973.

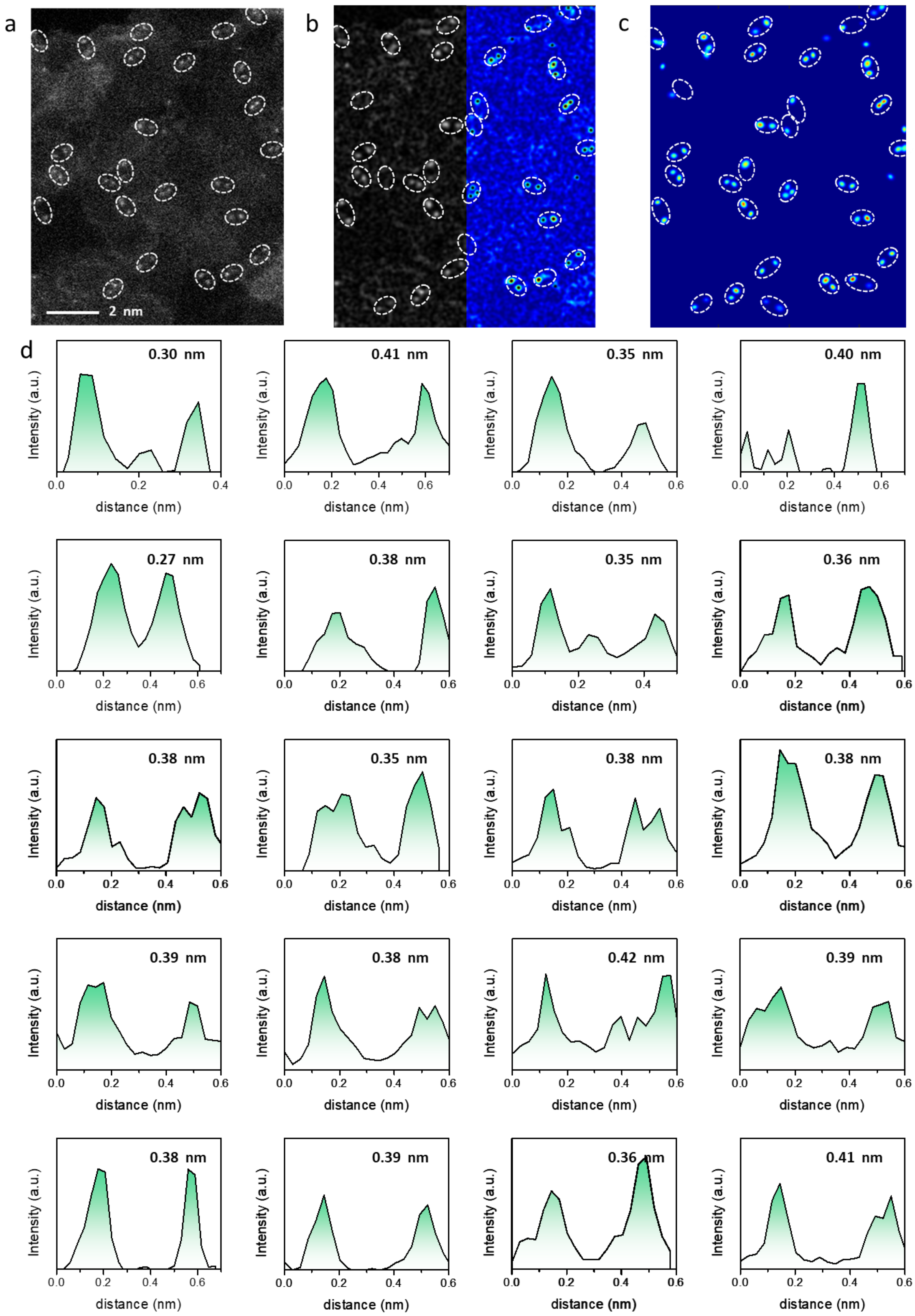

Supplementary Figure 12. (a) Representative HAADF-STEM image of Cu-NOC-973 and corresponding (b) 3D atom-overlapping Gaussian-function fitting maps; (c) fitting maps of Cu ICPs; (d) The corresponding profiles in (a).

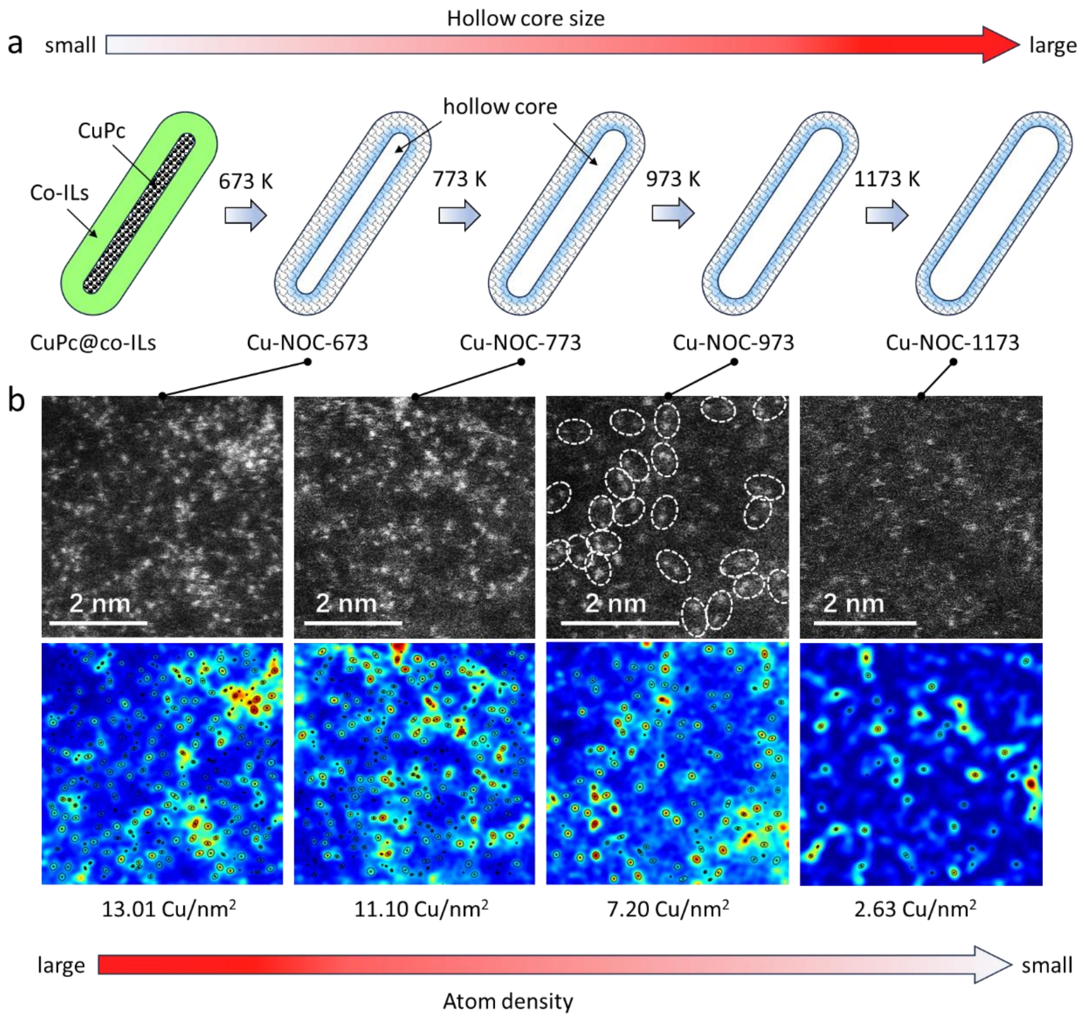

Supplementary Figure 13. (a) Macrostructural evolution of Cu-NOC catalyst molding process during different temperatures; (b) HAADF-STEM image of Cu-NOC and corresponding 3D atom-overlapping Gaussian-function fitting maps.

The copper phthalocyanine molecules in CuPc nanorods induce the decomposition of co-ILs, releasing gases containing C, N, O, and S, thereby forming porous walls. Due to the unbalanced interdiffusion between the ionic liquids and CuPc molecules, Kirkendall voids form at the interface between the co-ILs core and the CuPc shell. As the Kirkendall effect progresses, the CuPc core gradually decomposes, resulting in a hollow nanocapsule structure. During this process, the migration of Cu species induced by the Kirkendall effect leads to the formation of various Cu ensembles. Differences in heat treatment temperatures result in varying degrees of Kirkendall migration, affecting the final aggregation density of Cu species. As shown in Supplementary Figure 13b, at temperatures below 973 K, Cu atoms exhibit an aggregated state with a density exceeding 10 Cu/nm^2. When the temperature exceeds 973 K, the Cu atomic density gradually decreases, with Cu atoms forming diatomic dispersions at 973 K and transitioning to a fully monoatomic state at 1173 K. The Kirkendall effect directly influences the migration extent of Cu atoms, ultimately determining their dispersion state.

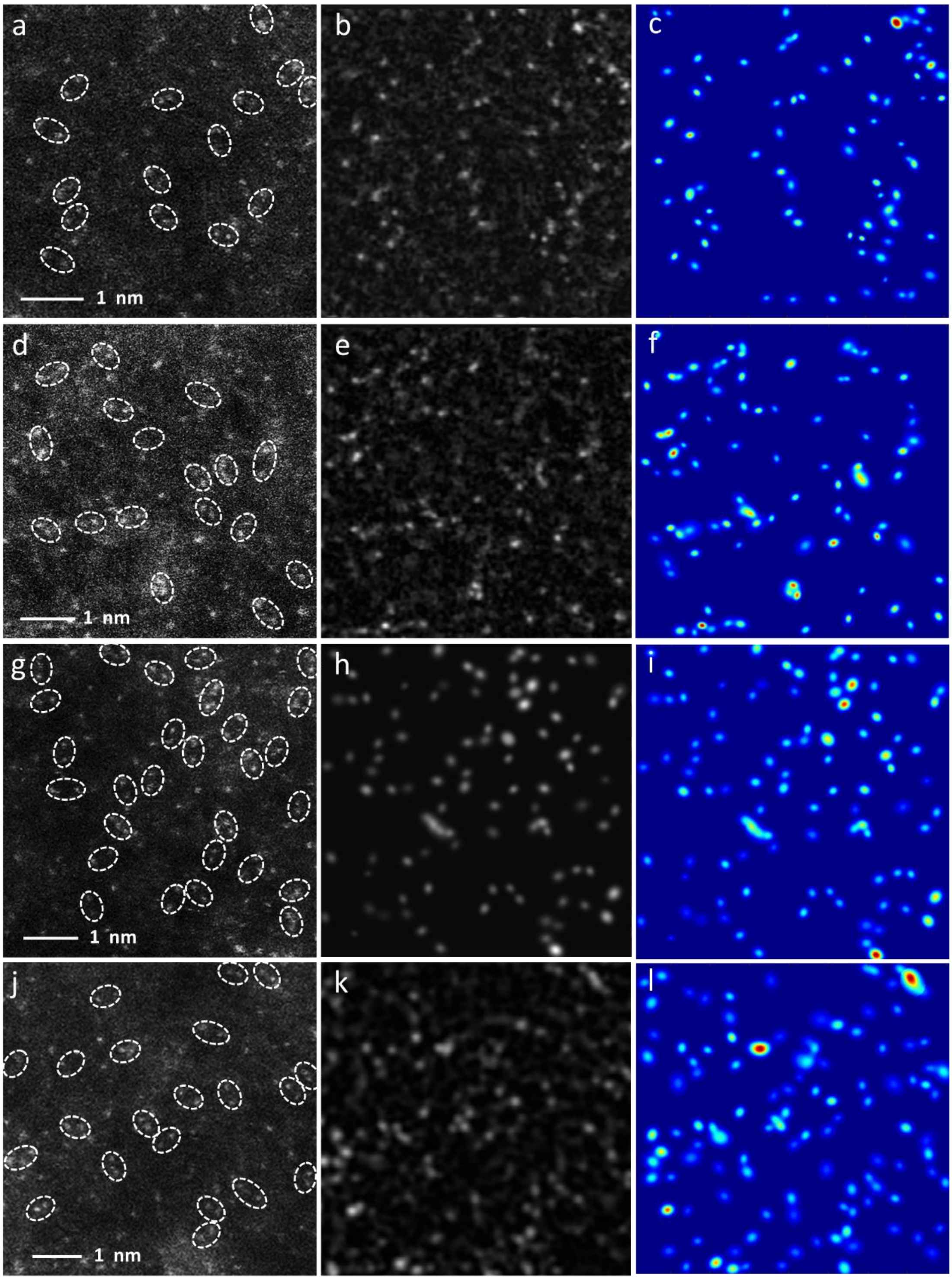

Supplementary Figure 14. HAADF-STEM image of and corresponding 3D atom-overlapping Gaussian-function fitting maps (a-c) Fe-NOC-973, (d-f) Co-NOC-973 , (g-i) Ni-NOC-973, and (j-l) Zn-NOC-973.

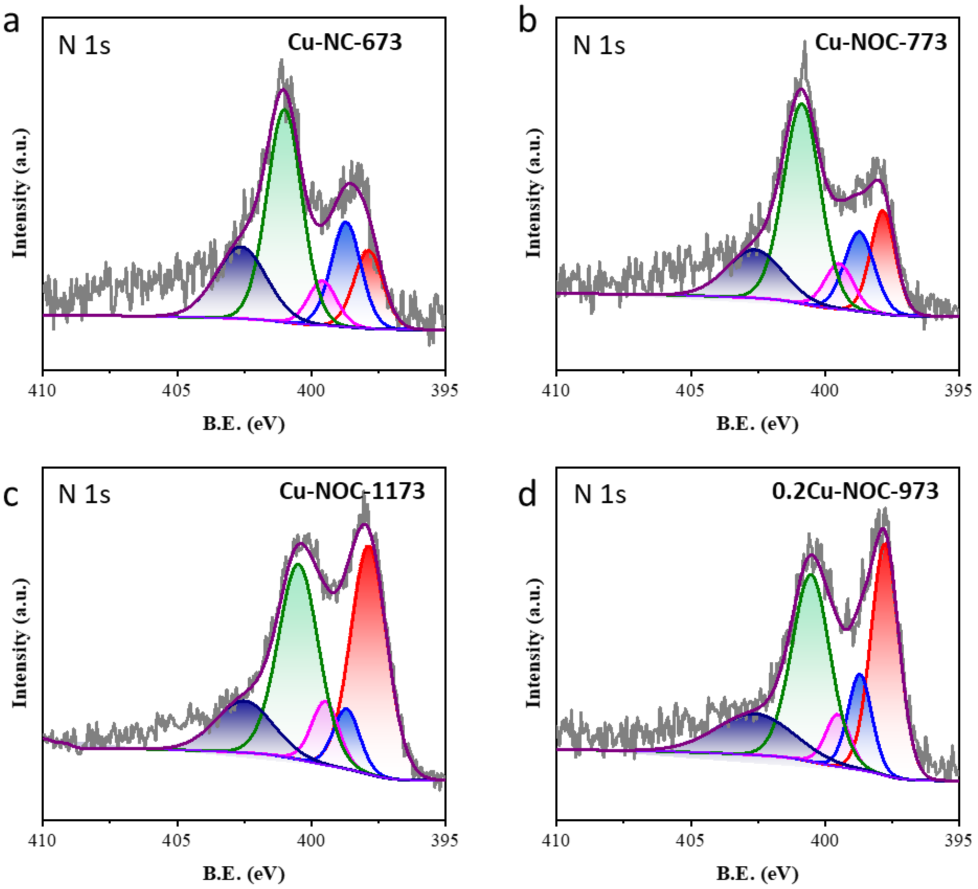

Supplementary Figure 15. The experimental and fitted high-resolution N 1s XPS spectra of (a) Cu-NOC-673, (b) Cu-NOC-773, (c) Cu-NOC-1173, and (d) 0.2Cu-NOC-973.

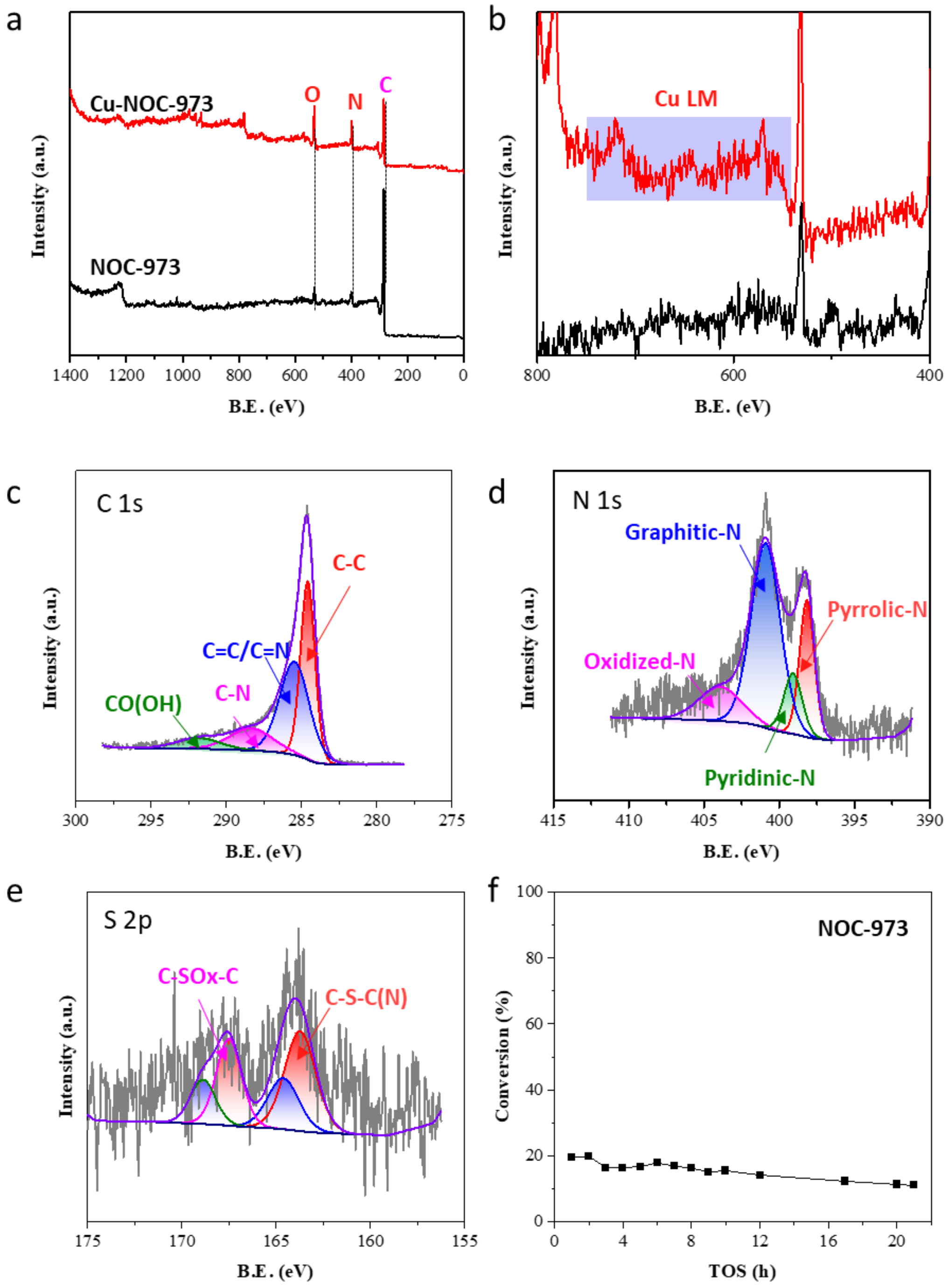

Supplementary Figure 16. (a)-(b) XPS survey spectrum of Cu-NOC-973 and NOC-973. The experimental and fitted high-resolution XPS spectra of (c) C 1*s*, (d) N 1*s*, and (e) S 2*p* for NOC-973; (f) Catalytic activity of NOC-973 at 473 K and GHSV(C_2H_2) = 50 h^{-1}.

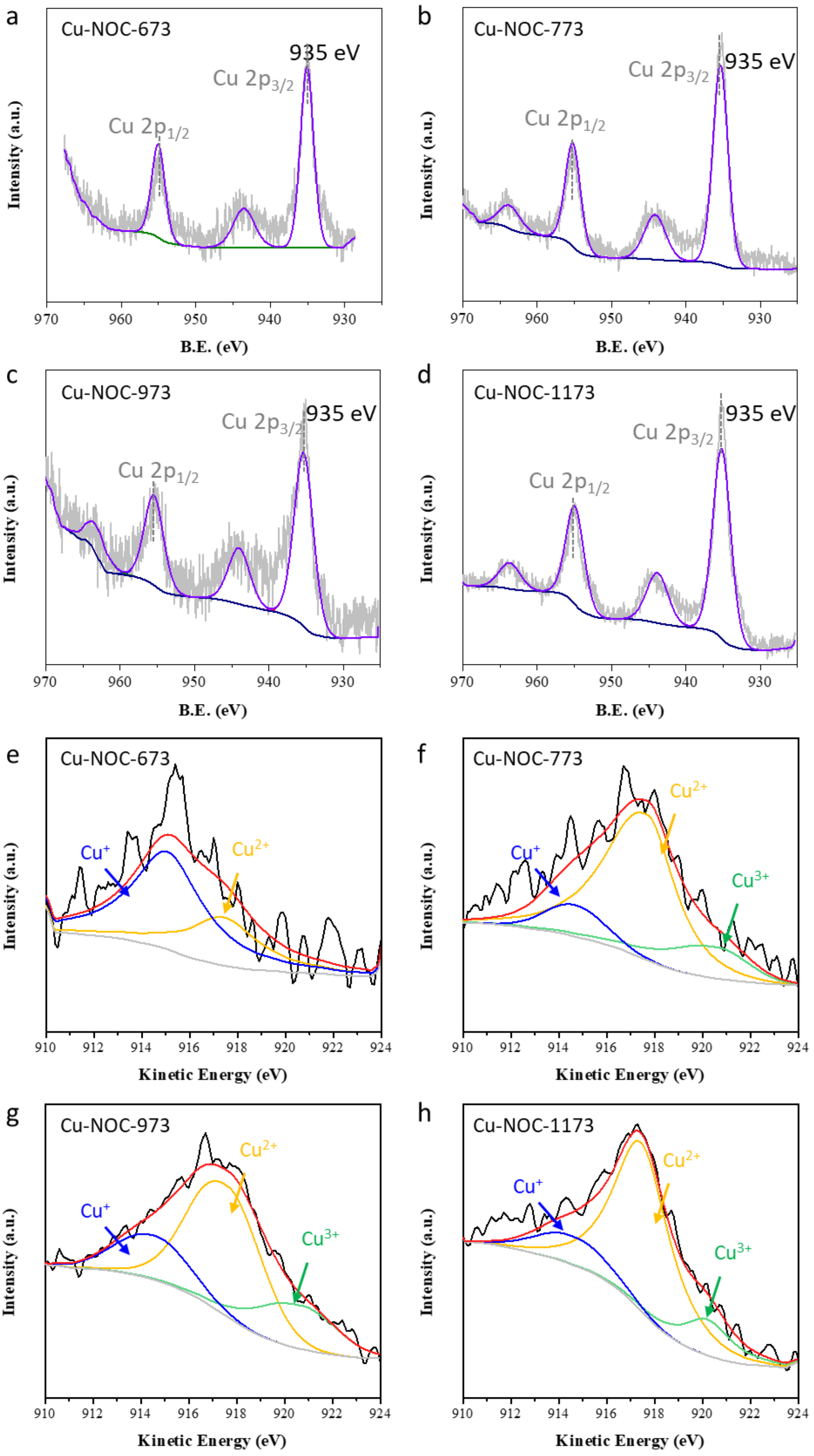

Supplementary Figure 17. Cu 2*p* XPS spectra of (a) Cu-NOC-673, (b) Cu-NOC-773, (c) Cu-NOC-973, and (d) Cu-NOC-1173 catalysts. Cu LMM Auger spectra of (e) Cu-NOC-673, (f) Cu-NOC-773, (g) Cu-NOC-973, and (h) Cu-NOC-1173 catalysts.

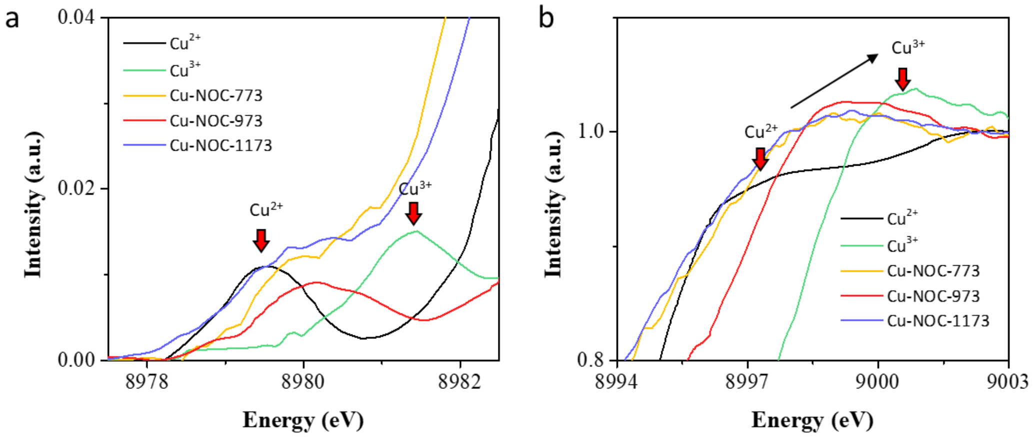

Supplementary Figure 18. (a) Cu *K*β HERFD-XAS pre-edge spectra of investigated samples; (b) The white line spectra of Cu *K*β HERFD-XAS pre-edge spectra for investigated samples. The Cu^{2+} and Cu^{3+} spectra were collected from Cu^{2+} and Cu^{3+} complexes in literature that reported by Liu *et al*.[7]

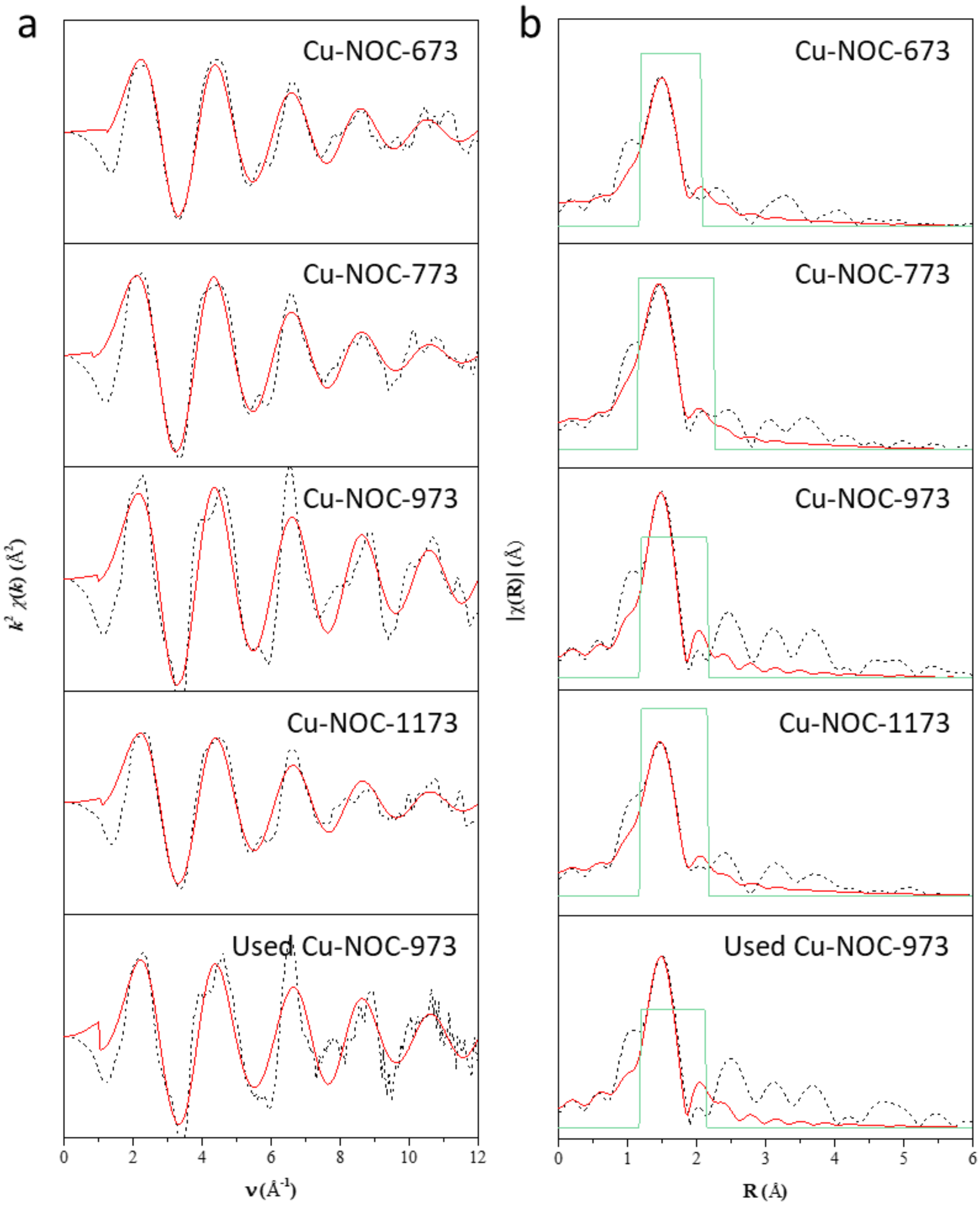

Supplementary Figure 19. Experimental and fitted EXAFS spectra at the Cu *K*-edge for selected copper catalysts. (a) *k*-space, and (b) *R*-space analysis of Cu-NOC-673, Cu-NOC-773, Cu-NOC-973, Cu-NOC-1173, respectively.

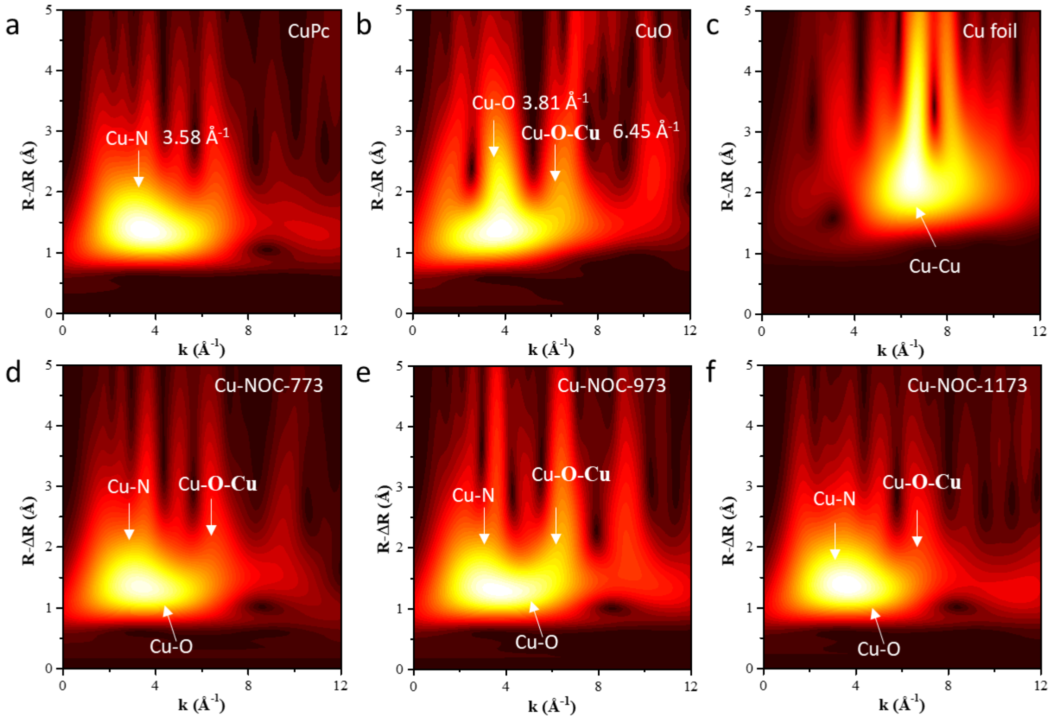

Supplementary Figure 20. Wavelet transforms (WT) of (a) CuPc, (b) CuO and (c) Cu foil, (d) Cu-NOC-773, (e) Cu-NOC-973 and (f) Cu-NOC-1173.

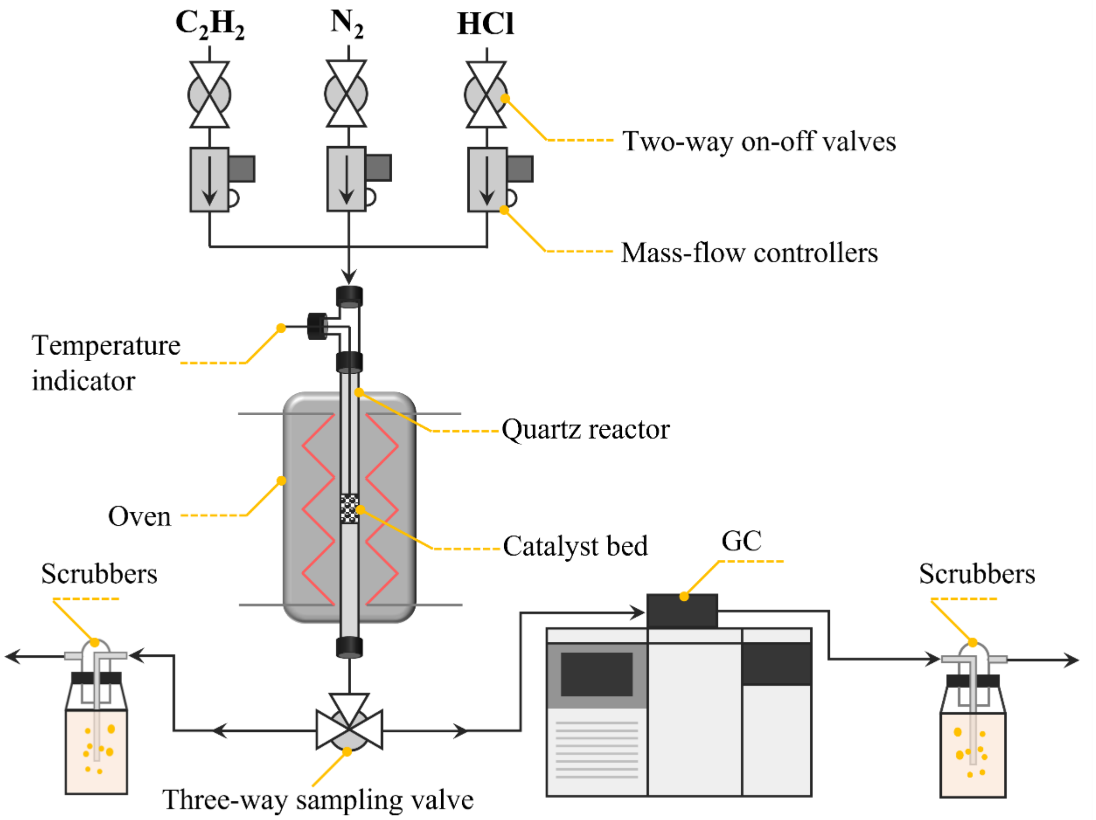

Supplementary Figure 21. Scheme of the laboratory set-up used for acetylene hydrochlorination.

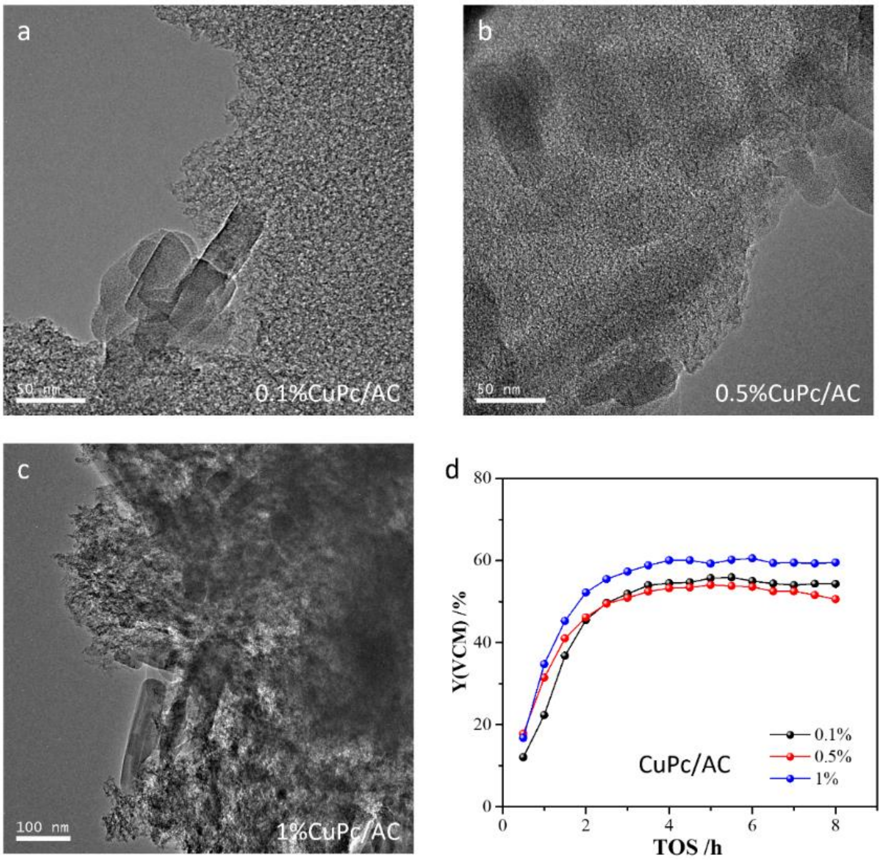

Supplementary Figure 22. TEM images of (a) 0.1CuPc/AC, (b) 0.5CuPc/AC and (c) 1%CuPc/AC catalysts; (d) Corresponding catalytic activity at 473 K and GHSV(C_2H_2) = 50 h^{-1}. 0.1, 0.5 and 1% represent the mass loading of copper in catalysts.

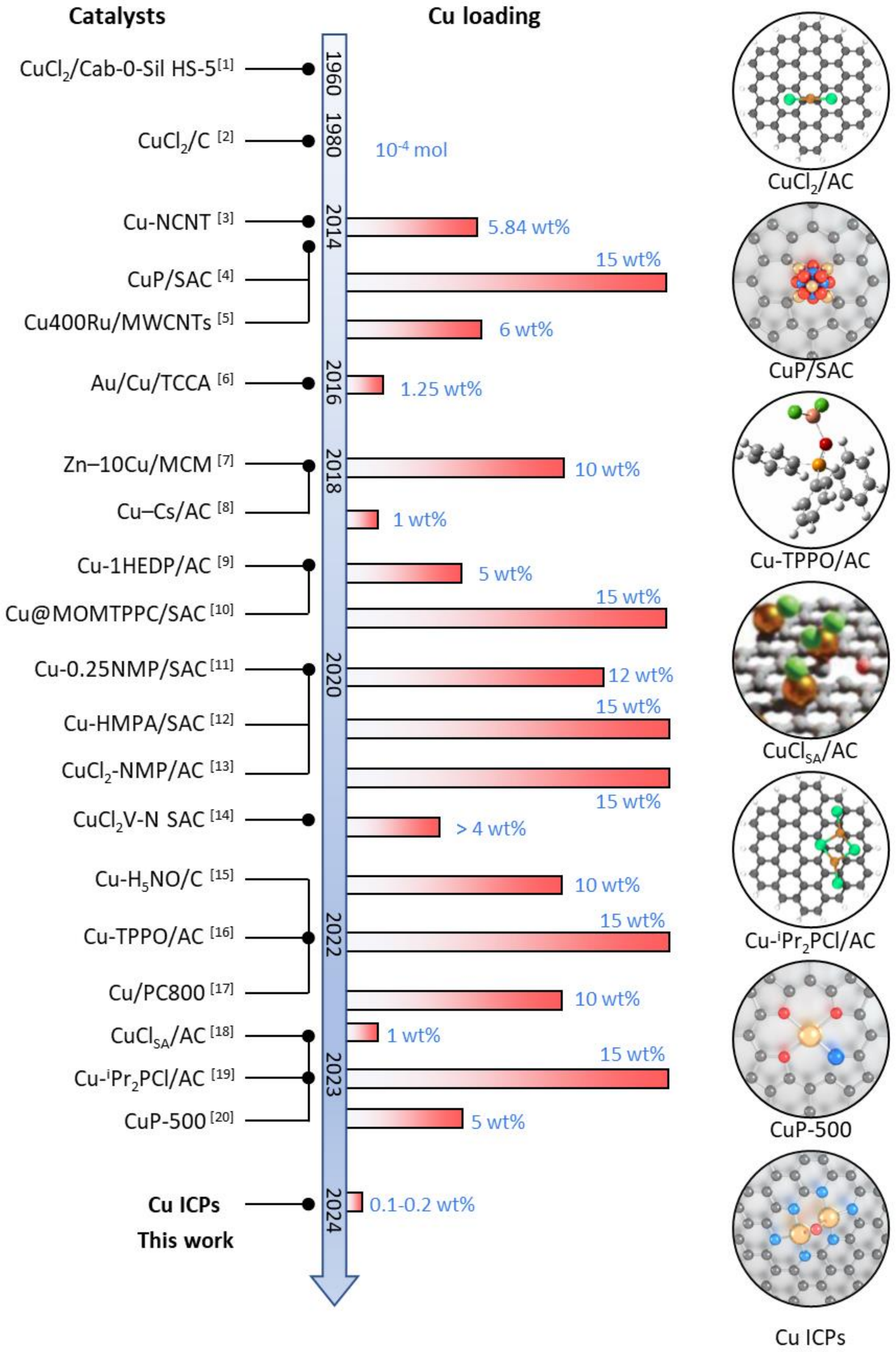

Supplementary Figure 23. Survey of acetylene hydrochlorination copper catalysts.

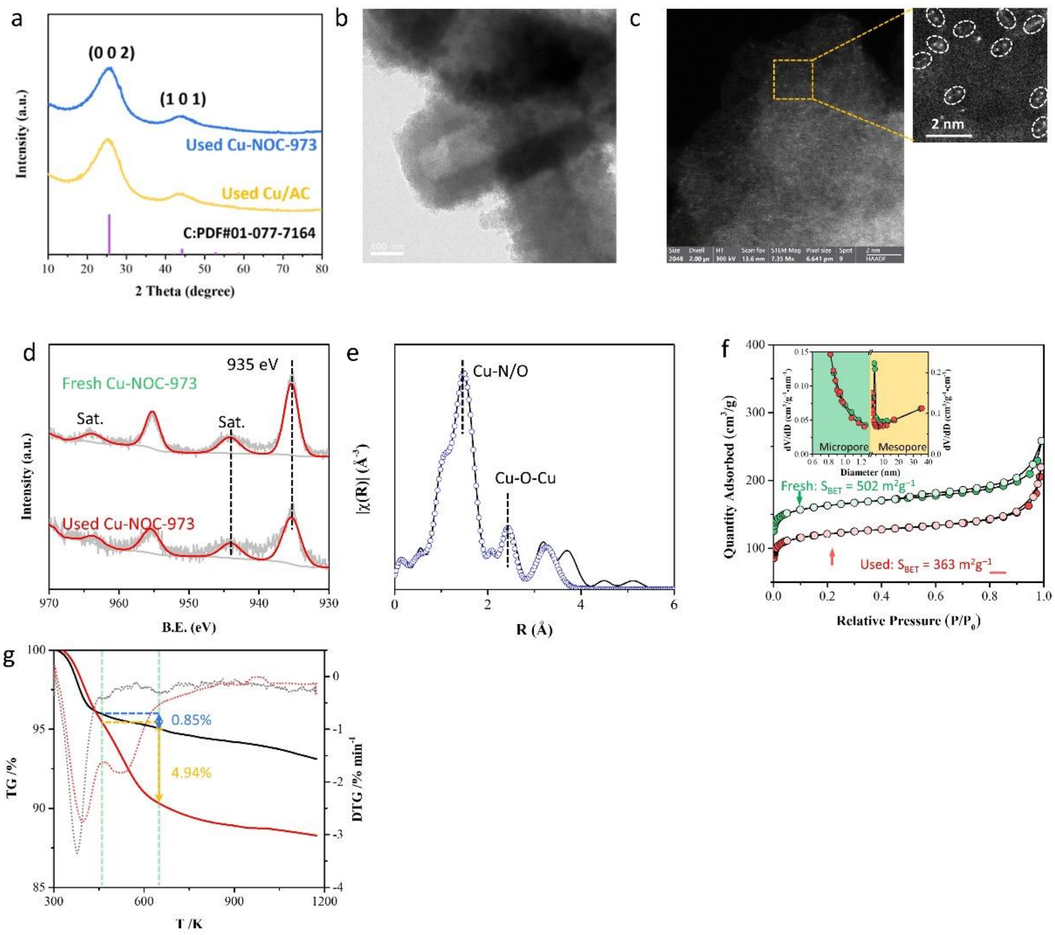

Supplementary Figure 24. (a) XRD patterns, (b) TEM image, and (c) HAADF-STEM images of used Cu-NOC-973; (d) Cu 2*p* XPS and (e) *R*-space spectra, (f) BET and (g) TG analysis of fresh and used Cu-NOC-973 catalysts.

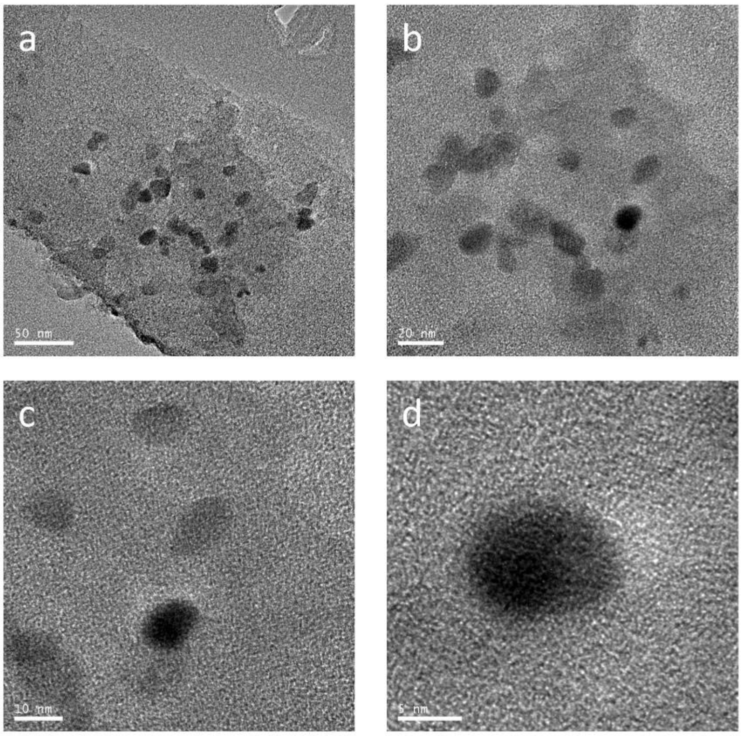

Supplementary Figure 25. (a)~(d) TEM images of used Cu/AC catalysts with Cu loading of 5 wt.%.

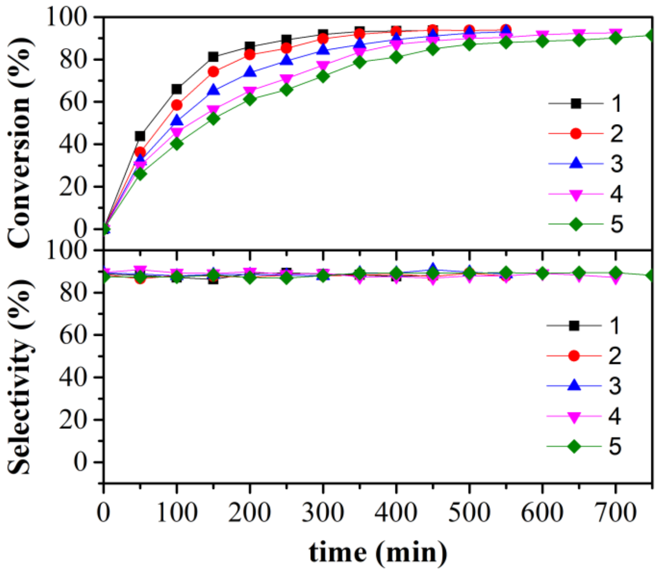

Supplementary Figure 26. Reusable cycles of Cu-NOC-973 for phenylpropyne hydrochlorination at 353 K.

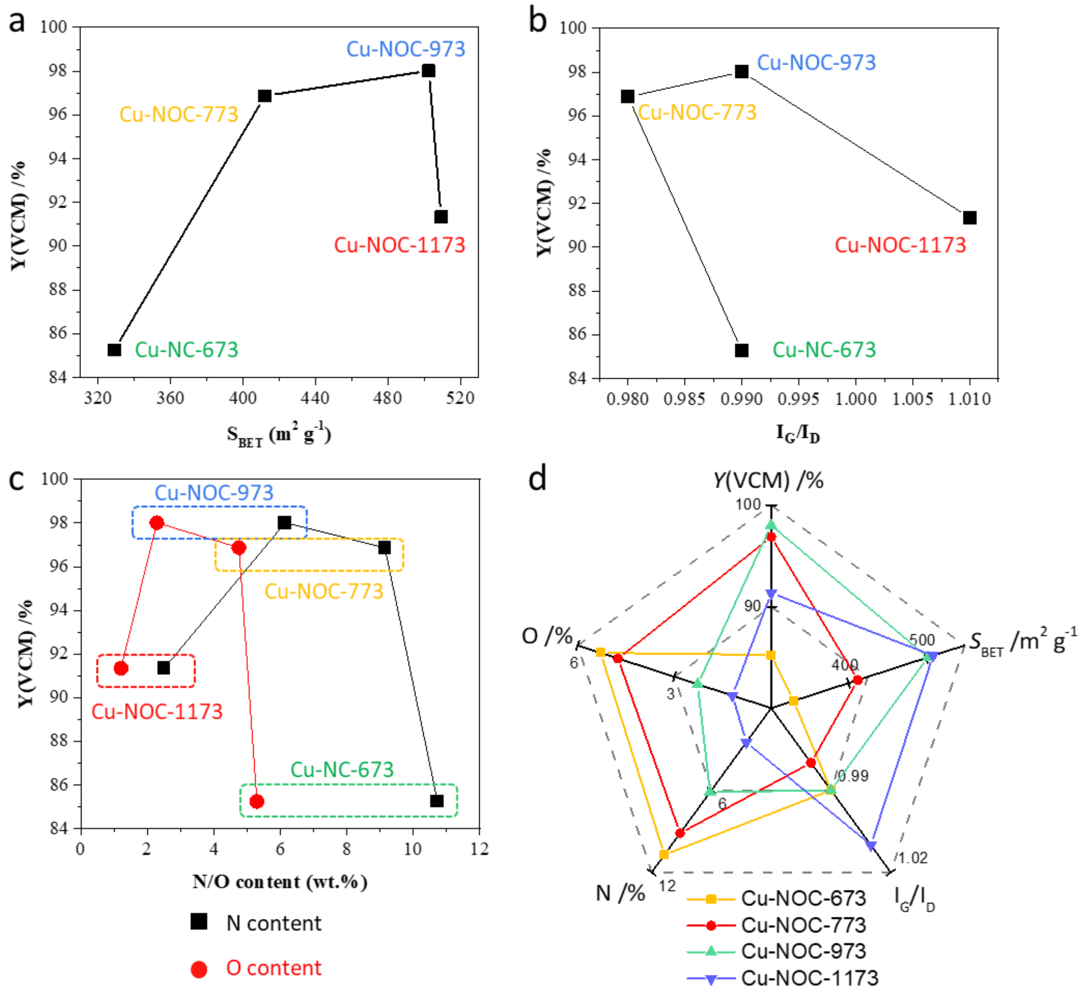

Supplementary Figure 27. Y(VCM) as a function of (a) surface area (m^2 g^{-1}), (b) I_G/I_D and (c) N/O content for Cu-NOC-673, Cu-NOC-773, Cu-NOC-973, and Cu-NOC-1173 catalysts; (d) radar plots.

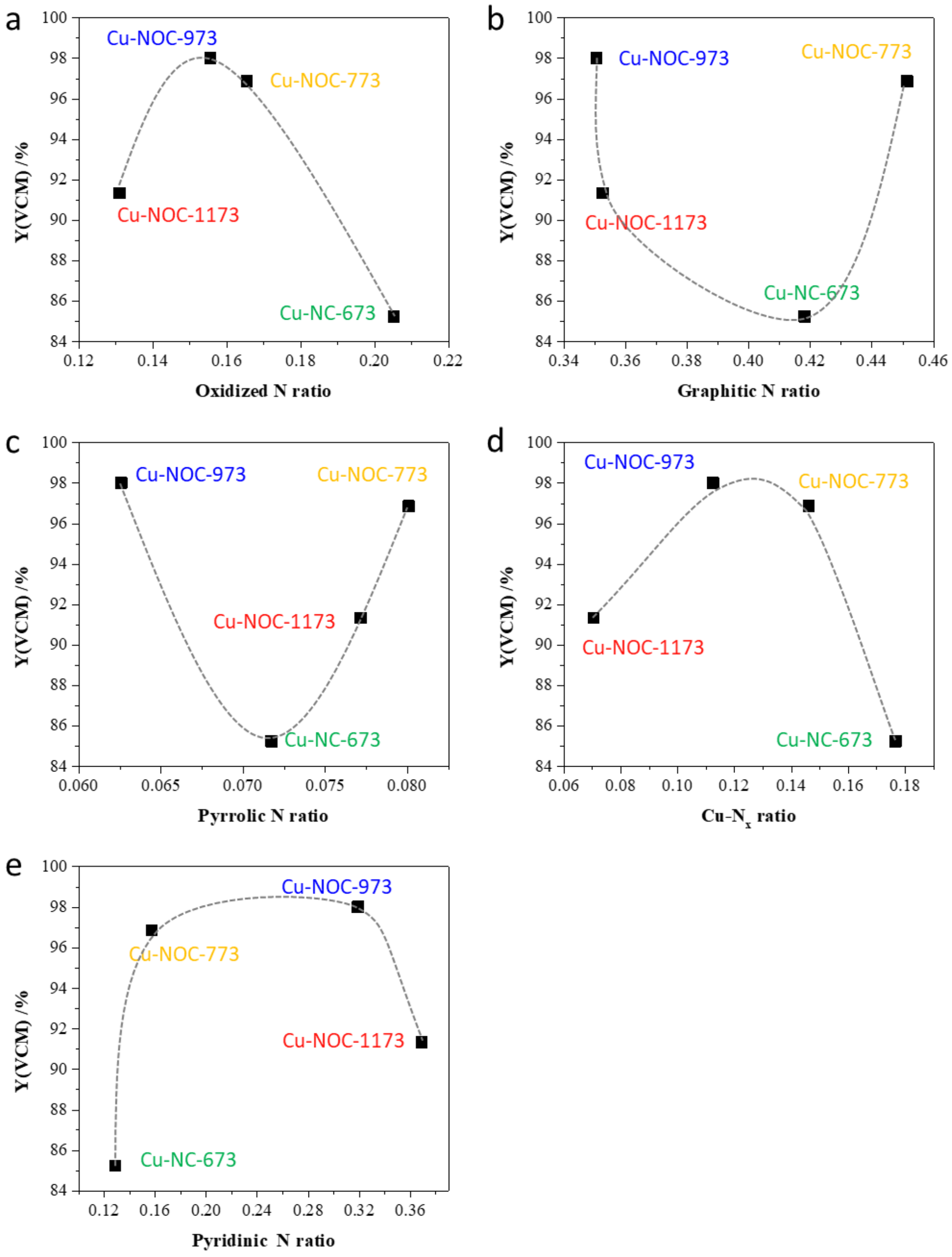

Supplementary Figure 28. Y(VCM) as a function of (a) oxidized N, (b) graphitic N, (c) pyrrolic N, (d) Cu-N_x and (e) pyridinic N ratio for Cu-NOC-673, Cu-NOC-773, Cu-NOC-973, and Cu-NOC-1173 catalysts.

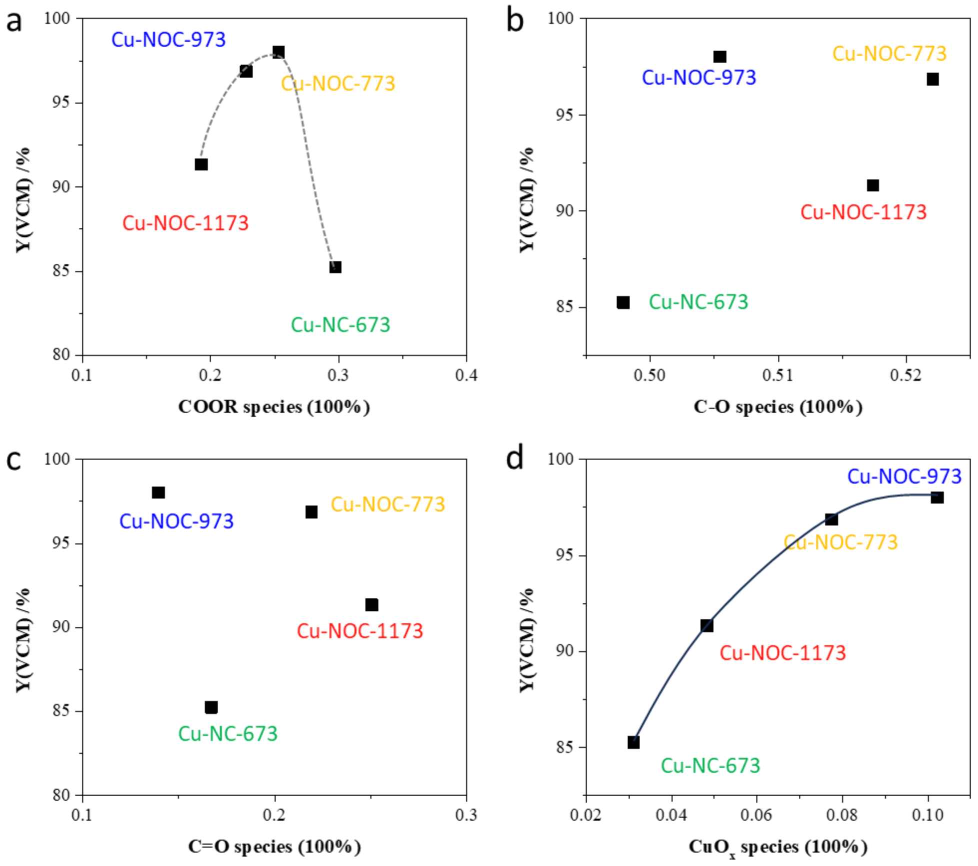

Supplementary Figure 29. Y(VCM) as a function of (a) COOR, (b) C-O, (c) C=O and (d) CuO_x species ratio for Cu-NOC-673, Cu-NOC-773, Cu-NOC-973, and Cu-NOC-1173 catalysts.

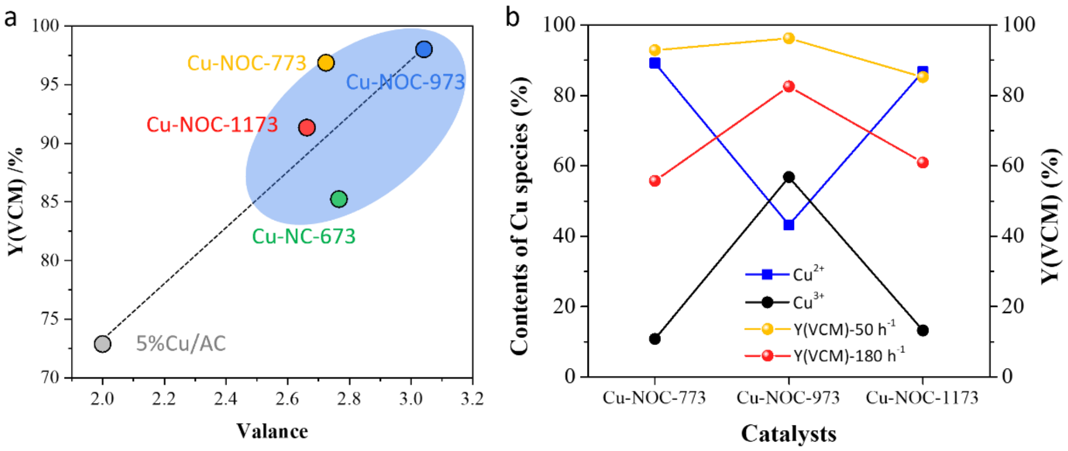

Supplementary Figure 30. (a) Y(VCM) as a function of specific Cu valance for Cu-NOC-673, Cu-NOC-773, Cu-NOC-973, and Cu-NOC-1173 catalysts; (b) Y(VCM) as a function of Cu^{2+} and Cu^{3+} contents, reaction condition: T = 473 K, GHSV(C_2H_2) = 50-180 h^{-1}.

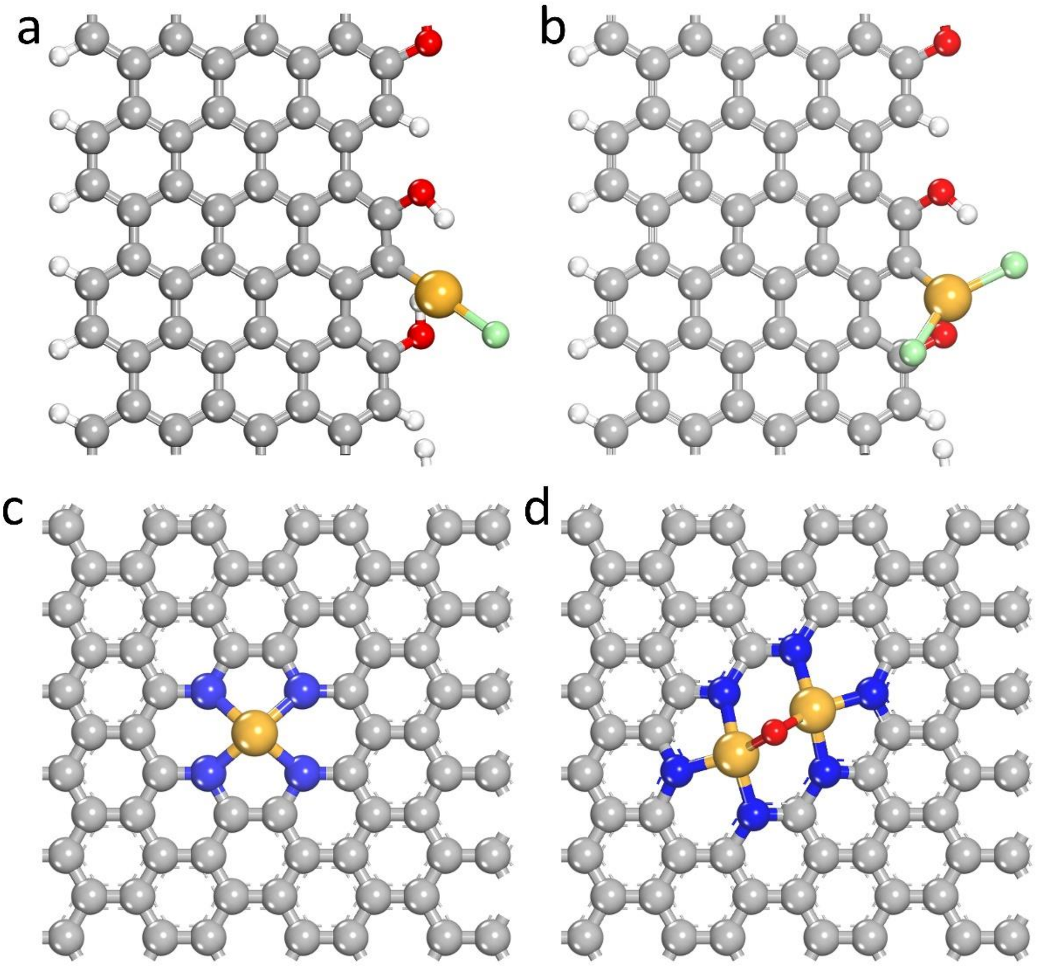

Supplementary Figure 31. Computational model of (a) CuCl, (b) $CuCl_2$,(c) CuN_4 and (d) CuN_3-O-CuN_3 ICPs.

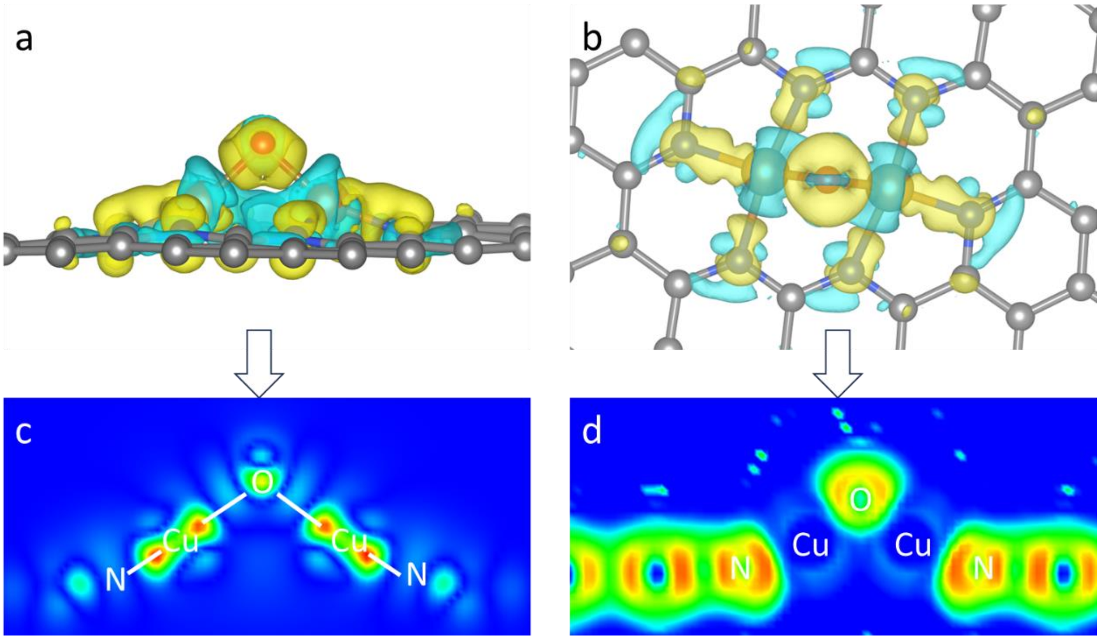

Supplementary Figure 32. (a&c) Charge density differences (Isosurface = 0.02) and (b&d) ELF of CuN_3-O-CuN_3 ICPs structures.

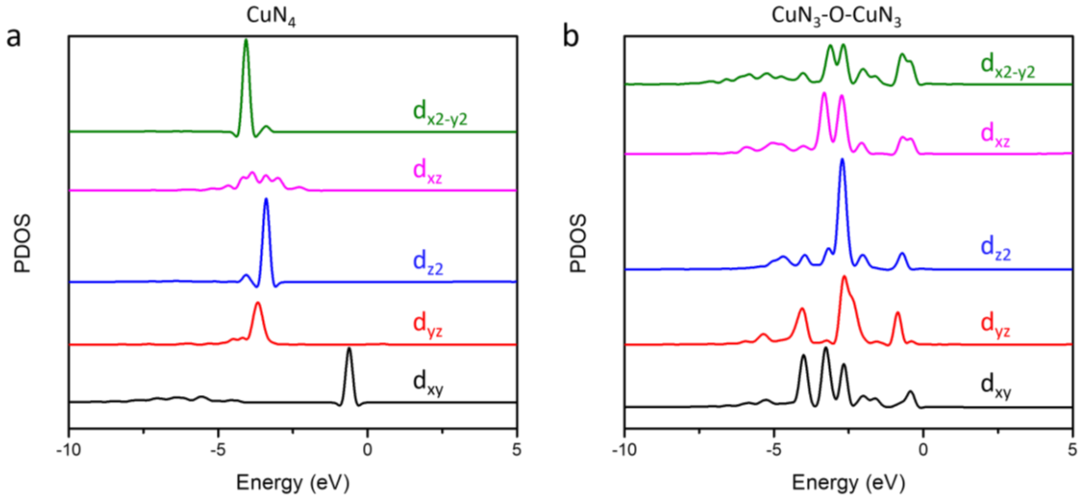

Supplementary Figure 33. Density of states of Cu 3*d* for (a) CuN_4 and (b) CuN_3-O-CuN_3.

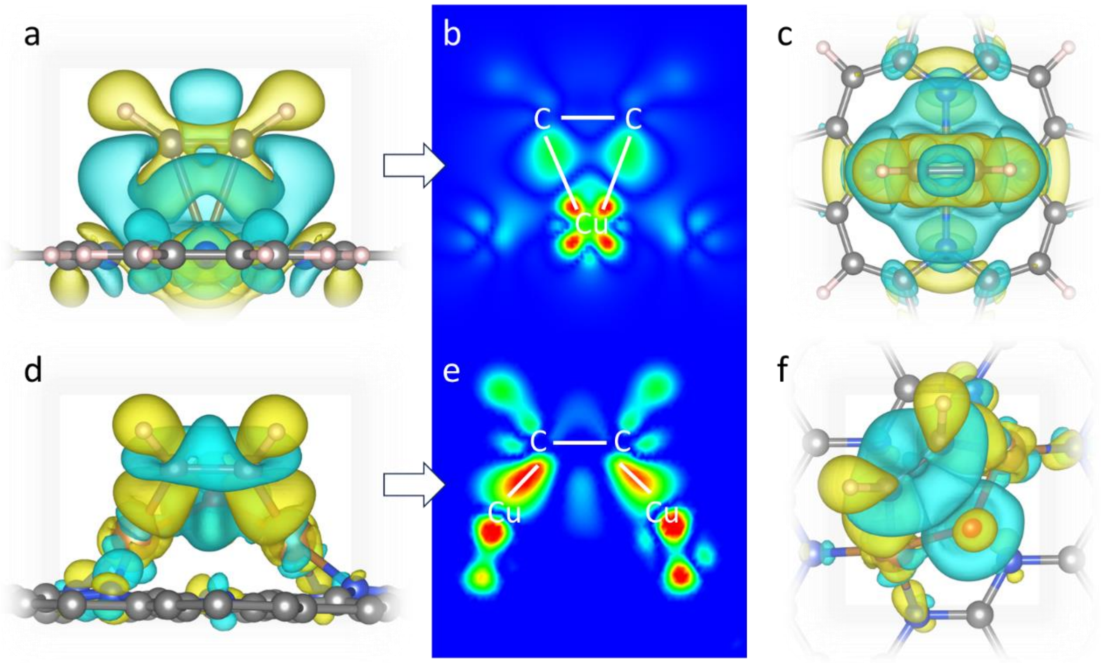

Supplementary Figure 34. Charge density differences of C_2H_2 adsorption on (a-c) CuN_4 sites and (d-f) CuN_3-O-CuN_3 ICPs (Isosurface = 0.02). (a&d) Front side view and (b&e) corresponding 2D slice images; (c&f) top side view.

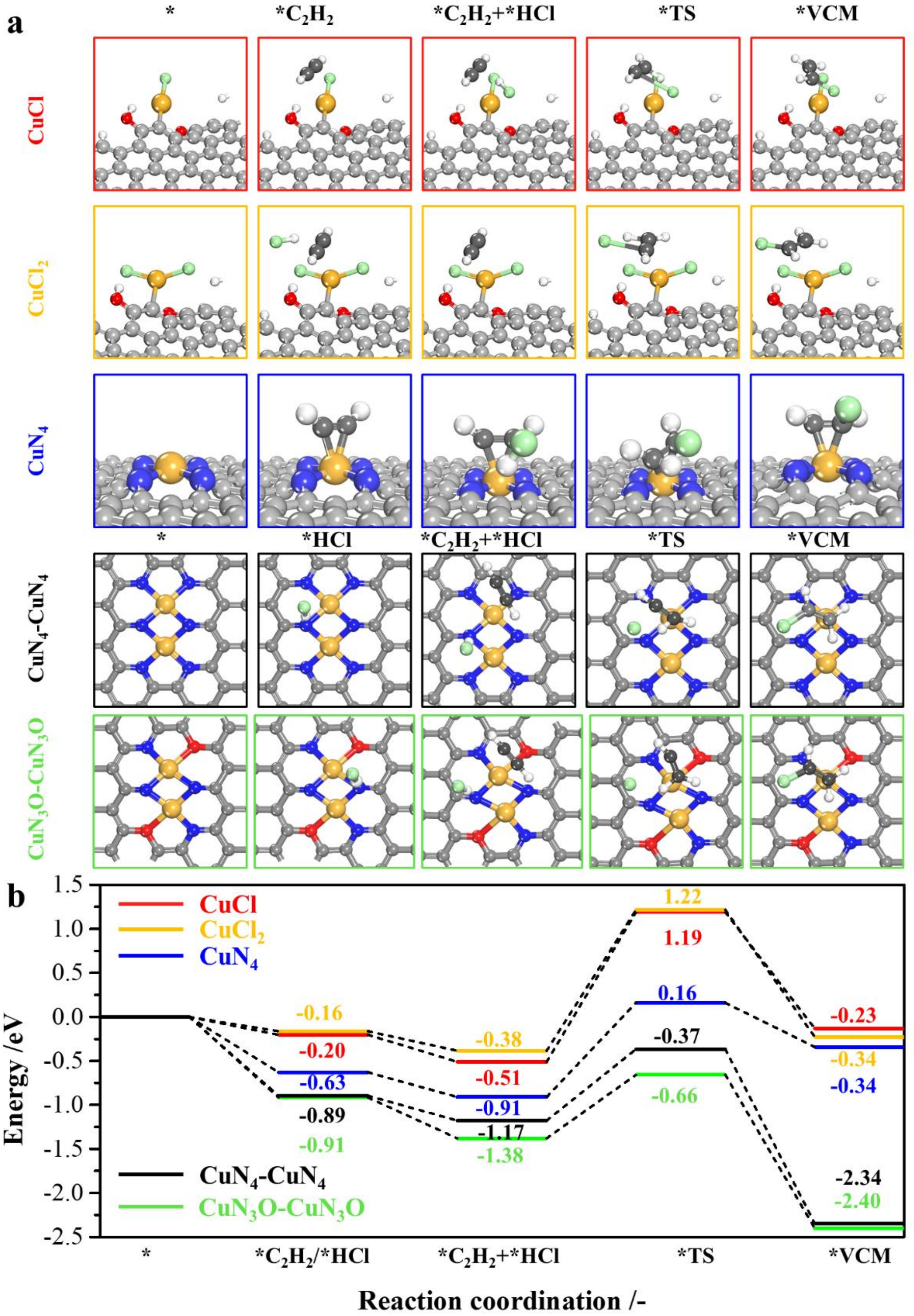

Supplementary Figure 35. (a) The reaction mechanism of acetylene hydrochlorination over investigated structures and corresponding (b) calculated potential energy surface.

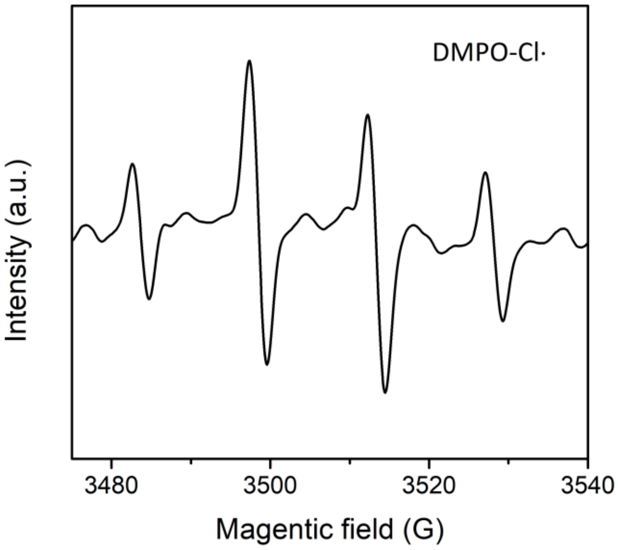

Supplementary Figure 36. ESR spectra of DMPO capturing Cl• generated by Bruker A300.

The EPR spin trapping technique was performed to confirm the generation of Cl• by Cu-NOC-973. DMPO was employed as the trapping agent of Cl•. As shown, a seven-line EPR spectrum of DMPOX appeared, which is identified as the oxidized derivative of DMPO via Cl• oxidation, intuitively showing the generation of Cl• after HCl adsorption on Cu-NOC-973.

Supplementary Table 1. The contents of the Cu, C, N, and O elements in as-prepared catalysts.

Catalysts	Elemental content (wt%)				
	C	N	O	S	Cu
NOC-973	82.27	9.59	7.73	0.41	/
Cu-NOC-673	83.72	10.72	5.29	0.19	0.08
Cu-NOC-773	85.95	9.14	4.75	0.05	0.11
Cu-NOC-973	91.46	6.14	2.28	/	0.12
Cu-NOC-1173	96.16	2.50	1.20	/	0.14
0.01Cu-NOC-973	92.02	5.14	2.82	/	0.013
0.05Cu-NOC-973	92.74	4.22	2.96	0.03	0.047
0.2Cu-NOC-973	92.77	4.52	2.52	/	0.19

Supplementary Table 2. BET and Raman results of investigated catalysts.

Sample	S_{BET} (m^2 g^{-1}) [a]	Volume (cm^3 g^{-1}) [b]	Diameter (nm) [c]	I_G/I_D
NOC-973	123.42	0.04	2.74	1.14
Cu-NOC-673	328.88	0.14	2.74	0.99
Cu-NOC-773	412.01	0.18	3.05	0.98
Cu-NOC-973	502.35	0.21	3.05	0.99
Cu-NOC-1173	509.20	0.23	2.75	1.01
0.01Cu-NOC-973	473.60	0.21	3.05	0.95
0.05Cu-NOC-973	456.70	0.18	3.06	0.96
0.2Cu-NOC-973	467.78	0.20	2.73	0.97
Used Cu-NOC-673	248.61	0.10	3.05	0.99
Used Cu-NOC-773	231.37	0.09	2.75	1.01
Used Cu-NPC-973	362.94	0.17	3.05	0.94
Used Cu-NPC-1173	385.25	0.17	3.06	1.07

Supplementary Table 3. LCF results of Cu-NOC series catalysts from Cu *K*β HERFD-XAS.

Catalysts	Weight (%)		R factor
	Cu^{2+}	Cu^{3+}	
Cu-NOC-773	89.2	10.8	0.045
Cu-NOC-973	43.2	56.8	0.053
Cu-NOC-1173	86.8	13.2	0.032

Linear combination fitting (LCF) calculations and additional micro-XANES measurements were performed. LCF analysis shows that XANES spectra of Cu-NOC samples can be completely described by a linear combination of the two reference XANES profiles: Cu^{2+} and Cu^{3+} complex that has been reported by Liu and Geoghegan *et al.*[7-8] In the Cu-NOC-973, 43.2% Cu species are incorporated in the Cu^{2+}, and 56.8% are in the form of Cu^{3+}, while in Cu-NOC-773 and Cu-NOC-1173, only a minor amount (below 15%) of Cu species is in the form of Cu^{3+}, and the majority (85%) are incorporated in the Cu^{2+}, further validating the coexistence of mixed-valence Cu^{2+}-Cu^{3+} species.

Supplementary Table 4. EXAFS parameters fitted for the samples.

Samples	Shell	CN[a]	R (Å)[b]	σ^2 (Å^2)	R-factor[c]
Cu foil	Cu-Cu	12.0	2.538	0.008	0.004
Cu-NPC-773	Cu-N<br>Cu-O	3.57<br>0.54	1.912<br>1.922	0.006	0.011
Cu-NPC-973	Cu-N<br>Cu-O	3.03<br>0.93	1.908<br>1.924	0.007	0.019
Cu-NPC-1173	Cu-N<br>Cu-O	2.95<br>0.56	1.924<br>1.922	0.007	0.007
Cu-NPC-973[d] (473 K, 740h^{-1})	Cu-N<br>Cu-O	3.05<br>0.94	1.907<br>1.923	0.004	0.015
Cu-NPC-973[e] (1173 K, 2h)	Cu-N<br>Cu-O	3.01<br>0.63	1.911<br>1.923	0.002	0.021
0.2Cu-NPC-973	Cu-N<br>Cu-O	3.07<br>0.87	1.906<br>1.923	0.003	0.017
CuPc	Cu-N	4.06	1.941	0.002	0.018

[a] Coordination number (CN)

[b] Atomic distance (R)

[c] Debye-Waller factor (R-factor)

[d] Reacted at 473 K and 740 h^{-1} for 8 h on stream

[e] Calcined at 1173 K under nitrogen atmosphere for 2 h

Supplementary Table 5. Catalytic performance of reported outstanding metal catalysts for acetylene hydrochlorination.

Catalysts	Cu loading /Wt%	T /K	GHSV /h^{-1}	Y(VCM)[b]	Ref.
$CuCl_2$/Cab-0-Sil HS-5	n.a.[a]	473	150	76	9
$CuCl_2$/C	10^{-4} mol	453	540	n.a.[a]	10
Cu-NCNT	5.84	453	180	45.8	11
CuP/SAC	15	413	180	72.4	12
Cu400Ru/MWCNTs	6	453	180	51.6	13
Au/Cu/TCCA	1.25	453	30	100	14
Zn–10Cu/MCM	10	533	90	98	15
Cu–Cs/AC	1	473	50	92	16
Cu-1HEDP/AC	5	453	90	83.4	17
Cu@MOMTPPC/SAC	15	453	180	92.2	18
Cu-0.25NMP/SAC	12	453	180	94.2	19
15 %Cu10 %HMPA/SAC	15	453	180	87.25	20
$CuCl_2$-NMP/AC	15	453	160	89	21
$CuCl_2$V-N SAC	4	393	n.a.[a]	n.a.[a]	22
Cu-H_5NO/C	10	433	90	n.a.[a]	23
15%Cu7%TPPO/AC	15	453	180	88	24
Cu/PC800	10	423	90	83.1	25
$CuCl_{SA}$/AC	1	473	670	~35	26
Cu-iPr2PCl/AC	15	453	180	93.7	27
CuP-500	5	433	90	100	28
Au-SA/AC	1	473	650	90	27
Pt-SA/AC	1	473	650	45	29
Au/AC	1	453	870	24	30
Pd/AC	1	453	870	12	30
Pt/AC	1	453	870	2	30
Ru/AC	1	453	870	15	30
Au/C-Acetone	1	473	17600	21	31
Au/NC	0.5	473	650	74	32
Au-CeO_2/AC	1	453	720	98	33
Au-Sr/AC	1	453	762	88	34
Pt/AC-w-473	1	473	650	60	35
Ru@TPPB/AC	1	443	360	100	36
Ru-Co-Cu/SAC	0.1	443	180	99	37
Ru/NC-g	1	473	650	95	38
Pd-IL/AC	0.5	433	740	98.6	39

[a]not available.

[b]Selectivity to vinyl chloride, S(VCM) >99% unless indicated otherwise.

Supplementary Table 6. Comparative performance of Cu-NOC-973 catalyst in alkynes hydrochlorination.

Reactants	Products	Temperature /°C	Time /h	Conversion /%	Selectivity /%
propyne	1-Chloro-1-propene	70	0.8	87.52	89.30
propargyl bromide	1-Chloro-3-propene	100	0.5	82.39	96.89
1-butyne	1-Chloro-1-butyne	75	1.0	88.76	97.42
1-pentyne	1-Chloro-1-pentene	50	1.0	90.05	93.13
phenylacetylene	1-Chloro-1-phenylethylene	35	0.8	81.66	98.75
phenylpropyne	1-Chloro-1-phenylpropene	90	2.5	87.23	90.78
2-butyne	1-Chloro-1-butyne	78	1.5	78.15	90.33

Supplementary Table 7. Experimental fitting data of Cu-NOC-973 catalyst and DFT-calculated data of the CuN_3-O-CuN_3 model.

Samples	Bond type	Bond length (Å)	
		Experimental results*	Calculation results
Cu foil	Cu-Cu	2.538	2.560
Cu-NOC-973	Cu-O	1.924	2.013
	Cu-N	1.908	1.933
	Cu-O-Cu	2.933	2.935
CuO	Cu-O	1.932	2.090
	Cu-O-Cu	2.947	3.015
C_2H_2	C≡C	\	1.213
HCl	Cl-H	\	1.311
VCM	C=C	\	1.342
	C-Cl	\	1.760

* Based on EXAFS results.

Supplementary Table 8. The integral quantity of PDOS for CuN_4 square-planar and CuN_3-O-CuN_3 ICPs coordination.

Coordination	*d*-orbital splitting manner	Integral quantity
	$d_{x^2-y^2}$	2.05
	d_{z^2}	1.92
CuN_4	d_{xz}	1.89
	d_{yz}	1.89
	d_{xy}	1.95
	d_{xz}	1.92
	d_{yz}	1.92
CuN_3-O-CuN_3	d_{xy}	1.88
	$d_{x^2-y^2}$	1.97
	d_{z^2}	1.95

Supplementary Table 9. Reaction schemes and corresponding energies of acetylene hydrochlorination on CuCl, $CuCl_2$ and CuN_3-O-CuN_3 ICPs.

Steps	Energy /eV				
	CuCl	$CuCl_2$	CuN_4	CuN_3-O-CuN_3 ICPs	
				Path 1	Path 2
C_2H_2 adsorption	-0.20	-0.16	-0.63	-1.68	-1.68
C_2H_2+HCl co-adsorption	-0.51	-0.38	-0.91	-3.08	-3.08
Transition state1	1.69	1.60	0.16	-2.46	-2.71
Intermediates	/	/	/	-3.26	-3.09
Transition state 2	/	/	/	-2.16	-3.27
VCM desorption	-0.13	-0.23	-0.34	-3.99	-3.99

Supplementary References

1. Kresse, G. & Furthmüller, J. Efficiency of ab-initio Total Energy Calculations for Metals and Semiconductors Using a plane-wave Basis Set. *Comput. Mater. Sci.* **6**, 15–50 (1996).
2. Kresse, G. & Furthmüller, J. Efficient iterative schemes forab initiototal-energy calculations using a plane-wave basis set. *Phys. Rev. B* **54**, 11169–11186 (1996).
3. Perdew, J. P., Burke, K. & Ernzerhof, M. Generalized Gradient Approximation Made Simple. *Phys. Rev. Lett.* **77**, 3865–3868 (1996).
4. Grimme, S. Semiempirical GGA-type Density Functional Constructed with a long-range Dispersion Correction. *J. Comput. Chem.* **27**, 1787–1799 (2006).
5. Grimme, S., Antony, J., Ehrlich, S. & Krieg, H. A Consistent and Accurate Ab Initio Parametrization of Density Functional Dispersion Correction (DFT-D) for the 94 Elements H-Pu. *J. Chem. Phys.* **132**, 154104 (2010).
6. Blöchl, P. E. Projector augmented-wave Method. *Phys. Rev. B* **50**, 17953–17979 (1994).
7. Liu, Y. *et al.* An Adaptable N-Heterocyclic Carbene Macrocycle Hosting Copper in Three Oxidation States. *Angew. Chem. Int. Ed.* **59**, 5696–5705 (2020).
8. Geoghegan, B. L. *et al.* Combining Valence-to-Core X-ray Emission and Cu K-edge X-ray Absorption Spectroscopies to Experimentally Assess Oxidation State in Organometallic Cu(I)/(II)/(III) Complexes. *J. Am. Chem. Soc.* **144**, 2520–2534 (2022).
9. Smith, D. M., Walsh, P. M. & Slager, T. L. Studies of silica-supported metal chloride catalysts for the vapor-phase hydrochlorination of acetylene. *J. Catal.* **11**, 113–130 (1968).
10. Nkosi, B., Coville, N. J. & Hutchings, G. J. Vapour phase hydrochlorination of acetylene with group VIII and IB metal chloride catalysts. *Appl. Catal.* **43**, 33–39 (1988).
11. Zhou, K. *et al.* Reactivity enhancement of N-CNTs in green catalysis of C_2H_2 hydrochlorination by a Cu catalyst. *RSC Adv.* **4**, 7766–7769 (2014).

12. Li, H., Wang, F., Cai, W., Zhang, J. & Zhang, X. Hydrochlorination of acetylene using supported phosphorus-doped Cu-based catalysts. *Catal. Sci. Technol.* **5**, 5174–5184 (2015).

13. Xu, J. *et al.* Ultra-low Ru-promoted $CuCl_2$ as highly active catalyst for the hydrochlorination of acetylene. *RSC Adv.* **5**, 38159–38163 (2015).

14. Xu, H., Zhou, K., Si, J., Li, C. & Luo, G. A ligand coordination approach for high reaction stability of an Au–Cu bimetallic carbon-based catalyst in the acetylene hydrochlorination process. *Catal. Sci. Technol.* **6**, 1357–1366 (2016).

15. Wang, Q. *et al.* Zn–Cu bimetallic catalysts supported on pure silica MCM-41 for acetylene hydration reaction. *New J. Chem.* **42**, 6507–6514 (2018).

16. Zhai, Y. *et al.* Carbon-supported perovskite-like $CsCuCl_3$ nanoparticles: a highly active and cost-effective heterogeneous catalyst for the hydrochlorination of acetylene to vinyl chloride. *Catal. Sci. Technol.* **8**, 2901–2908 (2018).

17. Wang, X., Zhu, M. & Dai, B. Effect of Phosphorus Ligand on Cu-Based Catalysts for Acetylene Hydrochlorination. *ACS Sustainable Chem. Eng.* **7**, 6170–6177 (2019).

18. Wang, Y., Nian, Y., Zhang, J., Li, W. & Han, Y. MOMTPPC improved Cu-based heterogeneous catalyst with high efficiency for acetylene hydrochlorination. *Mol. Catal.* **479**, 110612–110622 (2019).

19. Han, Y. *et al.* Pyrrolidone ligand improved Cu-based catalysts with high performance for acetylene hydrochlorination. *Appl. Organomet. Chem.* **35**, e6066–e6078 (2020).

20. Hu, Y. *et al.* High performance of supported Cu-based catalysts modulated via phosphamide coordination in acetylene hydrochlorination. *Appl. Catal. A Gen.* **591**, 117408–117417 (2020).

21. Zhao, C., Zhang, X., He, Z., Guan, Q. & Li, W. Demystifying the mechanism of NMP ligands in promoting Cu-catalyzed acetylene hydrochlorination: insights from a density functional theory study. *Inorg. Chem. Front.* **7**, 3204–3216 (2020).

22. Wang, B. *et al.* Controllable Synthesis of Vacancy-Defect Cu Site and Its Catalysis for the Manufacture of Vinyl Chloride Monomer. *ACS Catal.* **11**, 11016–11028

(2021).

23. Wang, B. *et al.* Electron-deficient Cu site catalyzed acetylene hydrochlorination. *Green Energy Environ.* (2022) doi:https://doi.org/10.1016/j.gee.2022.01.005.
24. Wang, B. *et al.* Phosphine-oxide organic ligand improved Cu-based catalyst for acetylene hydrochlorination. *Appl. Catal. A Gen.* **630**, 118461–118470 (2022).
25. Wang, T. *et al.* Interactions between atomically dispersed copper and phosphorous species are key for the hydrochlorination of acetylene. *Commun. Chem.* **5**, 1–10 (2022).
26. Akl, D. F. *et al.* Reaction-Induced Formation of Stable Mononuclear Cu(I)Cl Species on Carbon for Low-Footprint Vinyl Chloride Production. *Adv. Mater.* **35**, 2211464 (2023).
27. Zhang, T. *et al.* Excess Copper Chloride Induces Active Sites over Cu-Ligand Catalysts for Acetylene Hydrochlorination. *ACS Catal.* **13**, 8307–8316 (2023).
28. Tailoring Asymmetric Cu-O-P Coupling Site by Carbothermal Shock Method for Efficient Vinyl Chloride Synthesis over Carbon Supported Cu Catalysts. *ACS Catal.* **13**, 9777–9791 (2023).
29. Kaiser, S. K. *et al.* Performance Descriptors of Nanostructured Metal Catalysts for Acetylene Hydrochlorination. *Nat. Nanotechnol.* **17**, 606–612 (2022).
30. Conte, M. *et al.* Hydrochlorination of acetylene using supported bimetallic Au-based catalysts. *J. Catal.* **257**, 190-198 (2008).
31. Sun, X. *et al.* Facile synthesis of precious-metal single-site catalysts using organic solvents. *Nat. Chem.* **12**, 560-567 (2020).
32. Kaiser, S. K. *et al.* Controlling the speciation and reactivity of carbon-supported gold nanostructures for catalysed acetylene hydrochlorination. *Chem. Sci.* **10**, 359-369 (2019).
33. Ye, L. *et al.* Self- regeneration of Au/CeO2 based catalysts with enhanced activity and ultrastability for acetylene hydrochlorination. *Nat. Commun.* **10**, 914 (2019).
34. Li, G., Li, W. & Zhang, J. Strontium promoted activated carbon-supported gold catalysts for nonmercury catalytic acetylene hydrochlorination. *Catal. Sci. Technol.* **6**, 3230-3237 (2016).

35. Kaiser, S. K. *et al.* Nanostructuring unlocks high performance of platinum single-atom catalysts for stable vinyl chloride production. *Nat. Catal.* **3**, 376-385 (2020).

36. Shang, S. *et al.* Highly efficient Ru@IL/AC to substitute mercuric catalyst for acetylene hydrochlorination. *ACS Catal.* **7**, 3510-3520 (2017).

37. Zhang, H. *et al.* Ru-Co(III)-Cu(II)/SAC catalyst for acetylene hydrochlorination. *Appl. Catal. B* **189**, 56-64 (2016).

38. Kaiser, S. K., Lin, R., Krumeich, F., Safonova, O. V. & Pérez-Ramírez, J. Preserved in a shell: high-performance graphene-confined ruthenium nanoparticles in acetylene hydrochlorination. *Angew. Chem. Int. Ed.* **58**, 12297-12304 (2019).

39. Zhao, J. *et al.* Supported Ionic liquid-palladium Catalyst for the Highly Effective Hydrochlorination of Acetylene. *Chem. Eng. J.* **360**, 38–46 (2019).
